# The TMBIM1-YBX1 axis orchestrates MDSC recruitment and immunosuppressive microenvironment in pancreatic cancer

**DOI:** 10.7150/thno.111180

**Published:** 2025-02-03

**Authors:** Xuhui Tong, Mingming Xiao, Jing Yang, Jin Xu, Wei Wang, Xianjun Yu, Si Shi

**Affiliations:** 1 Department of Pancreatic Surgery, Fudan University Shanghai Cancer Center, Shanghai 200032, China.; 2 Department of Oncology, Shanghai Medical College, Fudan University, Shanghai 200032, China.; 3 Shanghai Pancreatic Cancer Institute, Shanghai 200032, China.; 4 Shanghai Key Laboratory of Precision Medicine for Pancreatic Cancer, Shanghai 200032, China.; 5 Pancreatic Cancer Institute, Fudan University, Shanghai 200032, China.

**Keywords:** TMBIM1, pancreatic cancer, immune evasion, MDSC

## Abstract

**Background:** Pancreatic ductal adenocarcinoma (PDAC) is notorious for its profoundly immunosuppressive nature. The complex crosstalk between diverse immune cell types and heterogeneous tumor cell populations shapes this challenging tumor immune microenvironment (TIME). In this study, the role of transmembrane BAX inhibitor motif-containing 1 (TMBIM1) in modulating the TIME and its potential as a therapeutic target in PDAC were investigated.

**Methods:** RNA sequencing was used to assess differential gene expression between PANC-1 cells with TMBIM1 knockdown and control cells. Single-cell RNA sequencing further validated the role of TMBIM1 in modulating the expression of CCL2 and PD-L1. Mechanistic insights were gained through chromatin immunoprecipitation, ELISA, real-time quantitative PCR, and flow cytometry experiments. To evaluate the impact of TMBIM1 on immune cell dynamics, we employed an *in vitro* chemotaxis assay and an *in vivo* C57BL/6J mouse xenograft model to examine CD8^+^ T-cell activation and myeloid-derived suppressor cell (MDSC) infiltration. Additionally, the therapeutic potential of TMBIM1 knockdown combined with anti-PD-1 antibody treatment was investigated in PDAC animal models.

**Results:** TMBIM1 was significantly upregulated in pancreatic cancer tissues and cell lines, driving pancreatic cancer cell proliferation, growth, and migration both *in vitro* and *in vivo*. Elevated TMBIM1 expression induced high infiltration of MDSCs and fostered an immunosuppressive tumor microenvironment. Mechanistically, TMBIM1 binds to the transcription factor Y box binding protein 1 (YBX1), which in turn increases the affinity of YBX1 for the PD-L1 and CCL2 gene promoters. This interaction results in their upregulation, leading to increased MDSC infiltration, thereby facilitating the immunosuppressive TIME in PDAC. Notably, the combination of TMBIM1 knockdown with anti-PD-1 therapy had a more potent antitumor effect than anti-PD-1 therapy alone.

**Conclusions:** Our study reveals that the TMBIM1/YBX1 axis is a key driver of immune evasion in PDAC and shapes the immunosuppressive TIME through the upregulation of CCL2 and PD-L1 expression. These findings highlight TMBIM1 as a potential therapeutic target to sensitize PDAC to immunotherapy.

## Introduction

Pancreatic ductal adenocarcinoma (PDAC) remains among the most aggressive and deadly cancers, with a dismal 5-year overall survival (OS) rate of less than 10% 1, 2. The asymptomatic nature of early-stage PDAC often leads to late diagnosis, leaving surgical resection viable for only approximately 20% of patients at presentation 3, 4. Even among those who undergo surgery, over 80% experience disease recurrence 3. Conventional treatments, such as chemotherapy and radiotherapy, generally yield limited and transient benefits, often providing only partial remission or temporary disease stabilization in newly diagnosed patients 5. Recent breakthroughs in understanding the tumor immune microenvironment (TIME) have led to improvements in immunotherapies, particularly immune checkpoint blockade (ICB), revolutionizing treatment strategies in oncology 6. However, unlike their remarkable success in certain solid tumors, such as melanoma and lung cancer, ICB therapies have shown minimal efficacy in PDAC, with only a minority of patients deriving clinical benefit 7-9.

The remarkable resistance of PDAC to immunotherapies is notably unique from that of other malignancies 1. The tumor microenvironment (TME) in PDAC is largely dominated by myeloid cells and significantly lacks cytotoxic T lymphocytes (CTLs), which, if present, have low levels of activation markers; these characteristics render the PDAC TME immunologically "cold." This deficiency in robust preexisting T-cell immunity is a key factor in disease progression and the poor response to ICB therapies 7. Myeloid-derived suppressor cells (MDSCs), which include pathologically activated monocytes and immature neutrophils, play a central role in this immune suppression 10. Two main subtypes of MDSCs—monocytic (M-MDSCs) and polymorphonuclear (PMN-MDSCs)—have been extensively studied, although the precise characterization of these cells remains a topic of debate within the field 11. Despite their phenotypic differences, both subsets share key biochemical and functional characteristics. Immunosuppressive capability is the hallmark of MDSCs, and the primary mediators of T-cell suppression by MDSCs include arginase 1 (Arg1), reactive oxygen species, nitric oxide, and prostaglandin E2 12, 13. The migration of MDSCs is significantly influenced by the chemokine receptor CXCR1/2 and its ligands, CXCL1 and CXCL5 14. Additionally, recent studies have identified chemokine ligand 2 (CCL2) as another recruiter of MDSCs via interaction with CCR2, further contributing to T-cell inhibition 15-19. Although targeting MDSC recruitment has demonstrated potential in reducing tumor growth in mouse models, its effectiveness as a therapeutic strategy in PDAC patients remains unclear 20. Currently, SX-682, a powerful allosteric inhibitor targeting CXCR1/2, is being assessed in clinical trials for PDAC (NCT04477343), although the results are yet to be published. Similarly, a CCL2 inhibitor, pirfenidone (PFD), was used to treat C57BL/6 J mouse bladder orthotopic tumor models, which resulted in a reduced tumor burden compared with that of the group given phosphate-buffered saline 19. Additionally, the CCR2 antagonist PF-04136309 has shown promise in enhancing antitumor immunity, leading to reduced tumor growth and metastasis in murine models of pancreatic cancer 21.

As a member of the transmembrane BAX inhibitor motif-containing (TMBIM) superfamily, TMBIM1 functions as a calcium channel in late endosomes and lysosomes, where it serves as a potent inhibitor of BAX-induced cell death 22. Previous studies have shown that TMBIM1 is aberrantly expressed across multiple tumor types, contributing significantly to tumor development 23, 24. Nevertheless, its specific function in the progression of PDAC and the mechanisms involved remain largely uninvestigated.

Our research revealed that TMBIM1 expression is significantly upregulated in PDAC tissues and cell lines and that TMBIM1 expression is strongly correlated with unfavorable patient outcomes. Functional assays demonstrated that TMBIM1 promotes tumor cell proliferation and migration. Furthermore, elevated TMBIM1 expression was found to correlate with increased levels of PD-L1 and CCL2. Mechanistic studies confirmed that TMBIM1 binds to Y box binding protein 1 (YBX1), thereby amplifying the transcriptional activation of both CCL2 and PD-L1, which in turn facilitates MDSC recruitment and concurrently dampens antitumor immunity in PDAC. These findings reveal a previously unexplored mechanism underlying tumor progression and metastasis, highlighting the intricate crosstalk between tumor cells and the TME and identifying potential therapeutic targets for PDAC. Notably, combining TMBIM1 knockdown with anti-PD-1 therapy elicited a robust immune response against PDAC tumor cells.

## Methods

### Clinical patient samples and tissue microarray (TMA)

A cohort of 169 PDAC specimens was collected from individuals diagnosed with PDAC (with R0 margin) based on histopathological examination at the Fudan University Shanghai Cancer Center (FUSCC) between 2012 and 2017. The study was conducted following the approval of the Institutional Research Ethics Committee at FUSCC, and all patients provided written informed consent before participating in the research.

### Cell culture

Human PDAC cell lines, including PANC-1, Capan-1, and CFPAC1 were obtained from the American Type Culture Collection and verified through short tandem repeat (STR) profiling. Normal human pancreatic ductal cells (HPDE) were kindly provided by the Li Lab (Min Li, University of Oklahoma Health Sciences Center). The mouse Pan02 cell line was sourced from the National Infrastructure of Cell Line Resource. PANC-1, HPDE, and Pan02 cells were maintained in DMEM, while CFPAC-1 and Capan-1 cells were grown in IMDM. All culture media were supplemented with 10% fetal bovine serum, 100 U/mL penicillin, and 100 μg/mL streptomycin, and cells were incubated at 37°C in a humidified atmosphere with 5% CO_2_.

### Plasmids and transfection

The coding sequences of human TMBIM1, with an added Flag tag at the 3' end, were cloned into the lentiviral vector pLVX-IRES-Neo (Tsingke, China) to construct TMBIM1-Flag overexpression plasmids, which were then used to generate lentivirus-infected pancreatic cancer cells (Table S1). For TMBIM1 knockdown, the pLKO.1 puro vector was employed to generate stable knockdown cell lines using shRNA. The specific sequences for shTMBIM1 and YBX1 silencing (siYBX1) are listed in Table S1. Pancreatic cancer cells were transfected with siRNAs utilizing Lipofectamine 3000 reagent (Invitrogen, USA) following the manufacturer's protocol.

### Data download and bioinformatics analysis

This study utilized single-cell RNA sequencing data from human pancreatic cancer tissues obtained from two key databases: the Gene Expression Omnibus (GEO) with accession number GSE212966, and the Genome Sequence Archive under accession number CRA001160, accessible at https://bigd.big.ac.cn/bioproject/browse/PRJCA001063. The GSE212966 dataset comprised 12 samples, with an equal distribution of 6 pancreatic cancer tissues and 6 normal pancreatic tissues. In contrast, the CRA001160 dataset included 35 samples, of which 24 were pancreatic cancer tissues and 11 were normal tissues. Data processing involved constructing an expression matrix using the CellRanger software suite (10x Genomics). Quality control measures were applied to exclude low-quality barcodes and cells with minimal library sizes (fewer than 1000 UMIs) or limited gene expression profiles.

For bulk RNA sequencing analysis, data from the TCGA-PAAD (The Cancer Genome Atlas-Pancreatic Adenocarcinoma) cohort were utilized, focusing solely on tumor samples, while normal tissue samples were supplemented from the GTEx project due to the limited availability in the TCGA-PAAD dataset. Additionally, proteomics data from the CPTAC (Clinical Proteomic Tumor Analysis Consortium) were analyzed to provide a complementary perspective on protein expression patterns. The proportions of immune cell populations were estimated using TIMER 2.0 (http://timer.cistrome.org/) and validated with additional computational methods, including TIDE, XCELL, and CIBERSORT, all integrated into the TIMER 2.0 platform.

### RNA extraction and real-time quantitative PCR (qPCR)

We carried out the RNA extraction from cells and tissues utilizing the Total RNA Kit I (Accurate Biotechnology Co Ltd, China) following the manufacturer's protocol. The extracted RNA was then reverse-transcribed into cDNA using the HiScript III 1st Strand cDNA Synthesis Kit (Vazyme, China), ensuring high-quality cDNA synthesis. qPCR was performed using specific primers for β-Actin, TMBIM1, CCL2, PD-L1, Arg1, and inducible nitric oxide synthase (iNOS), along with the SYBR Green Supermix (Vazyme, China) on the StepOnePlus System (Applied Biosystems, USA). The qPCR reactions were conducted in triplicate for each experimental group to ensure reproducibility and accuracy of the results. Primer sequences utilized in these experiments are detailed in Table S2.

### RNA sequencing

RNA was extracted from PANC-1 shNC and PANC1 shTMBIM1 cells using TRIzol reagent (Sigma, USA), and each cell line was subjected to analysis in triplicate to enhance the reliability of the results. Subsequent RNA sequencing was carried out using the Illumina HiSeq 3000 Sequencing System. The fragments per kilobase of exon per million mapped reads (FPKM) for each gene were then calculated and analyzed, providing insights into gene expression levels across the samples.

### CCK-8 assay

Cell growth was quantified using the CCK-8 assay. A total of 3 × 10³ cells per well were seeded into 96-well plates. The assay was performed at 0, 24, 48, 72, and 96 hours after plating. The CCK-8 Cell Proliferation Assay Kit (Solarbio Science & Technology Co., Ltd., Beijing, China) was used according to the manufacturer's instructions. Absorbance at 450 nm was recorded with a microplate reader to determine cell proliferation.

### Edu assay

Cell proliferation was evaluated using the BeyoClick™ EdU-594 Kit (Beyotime, C0078S). Cells were seeded into 6-well plates at a density of 1 × 10⁵ cells per well and cultured for 24 hours. After 48 hours of proliferation, EdU was added to each well at a final concentration of 10 μM and incubated at 37°C for 4 hours to label proliferating cells. Subsequent steps were carried out in strict accordance with the protocol provided by the manufacturer.

### Transwell Migration Assay

The experiment utilized CoStar transwell chambers with a pore size of 8 μm. In this setup, 3 × 10^4^ cells per well were placed in the upper chamber in 200 µl of serum-free medium. The lower chamber was supplemented with 600 µl of medium containing 10% FBS to act as a chemoattractant and encourage cell migration. Following a 36-hour incubation period at 37 °C in an atmosphere containing 5% CO_2_, non-migrated cells remaining on the upper surface of the membrane were gently removed. For the fixation and staining process, migrated cells present on the lower surface of the membrane were fixed with 4% paraformaldehyde (PFA) for stability and then stained with 0.1% crystal violet to visualize the cells. This staining process lasted for 30 min, after which the cells were imaged and quantified under a microscope.

### Colony formation assay

In total, 7 × 10^2^ shRNA-transfected Capan-1 and PANC-1 cells were seeded in complete medium into 6-well plates and cultured at 37°C in a 5% CO2 atmosphere for 14 days. After incubation, the cells were fixed with 4% paraformaldehyde for 30 minutes and stained with 0.2% crystal violet solution (Beyotime, C0121). Captured images were and analyzed by online Image J platform (https://cnij.imjoy.io/).

### Western blotting

Cells were lysed on ice for 20 minutes with lysis buffer (Beyotime Biotechnology, China) containing protease and phosphatase inhibitors (Beyotime Biotechnology, China). Following this, the samples were centrifuged at 16,000g for 10 minutes to extract the proteins. The resulting protein lysates were separated by SDS-PAGE and subsequently transferred to nitrocellulose membranes (Millipore, USA). A complete list of the antibodies utilized in this study can be found in online Table S2.

### ELISA

The ELISA assay was conducted in accordance with the manufacturer's protocols, with titration adjustments made based on prior experimental procedures. Concentrations of CCL2 and PD-L1 in the supernatants of PDAC cell lines cultured under various conditions, as well as in tumor samples from the *in situ* pancreatic tumor model in mice, were quantified using ELISA. Tumors from mice were lysed with RIPA buffer, and total protein concentrations were assessed using a BCA assay. To ensure consistency, protein levels across samples were normalized using the lysis buffer prior to the ELISA analysis. A detailed list of the reagents utilized can be found in Table S4.

### Silver staining

The interacting proteins were detected using a silver staining kit, following the manufacturer's instructions (Thermo Fisher Scientific, USA).

### Protein-protein docking

The amino acid sequences for human TMBIM1 (ID: Q969X1) and YBX1 (ID: P67809) were retrieved from the UniProt database. Docking analyses of TMBIM1 and YBX1 were performed using HDOCK, and the resulting protein-protein interactions were visualized with PyMOL.

### Coimmunoprecipitation (co-IP) assay

To evaluate exogenous interactions, Flag-tagged TMBIM1 overexpression plasmids were transfected into CFPAC1 cells. Cell extracts were then incubated overnight at 4 °C with ChIP-Grade Protein A/G Magnetic beads (Thermo Fisher, USA) and Anti-Flag antibody (Sigma, F1802, USA). For the assessment of endogenous interactions, the extracts were treated with Protein A/G Magnetic beads along with either an IgG control or YBX1 antibody (Proteintech, 20339-1-AP, China). Afterward, the protein samples underwent three rounds of washing with IP buffer (Beyotime, China) before proceeding to Western blot analysis.

### Chromatin immunoprecipitation (ChIP)

ChIP Assay Kit (Thermo Fisher, USA) was utilized following the manufacturer's guidelines. In brief, cells from each group were cross-linked using a 1% formaldehyde solution. Afterward, the cells were harvested and resuspended in SDS lysis buffer, followed by sonication. The mixture was centrifuged to separate the cellular debris, and the supernatant was combined with ChIP dilution buffer. Subsequently, agarose beads and either an anti-YBX1 antibody (Santacruz, sc-101198, USA) or an anti-IgG antibody were added, followed by overnight incubation at 4 °C. After washing, the proteins were eluted from the beads and subjected to heating at 65 °C for 4 hours. Finally, the enrichment of YBX1 at the CCL2 and PD-L1 promoter regions was evaluated using qPCR, with the relevant primer sequences listed in Table S2.

### Luciferase reporter assay

The promoter regions of CCL2 and PD-L1 were amplified from genomic DNA, targeting the area from -2000 to +100 relative to the transcription start site, and then ligated into the pGL3-Basic vector. Following this, the dual-luciferase assay system (Vazyme, China) was utilized to assess both Renilla and firefly luciferase activities, adhering to the provided manufacturer's guidelines.

### Immunohistochemistry (IHC)

The processing of paraffin-embedded tissue slides involved several key steps: first, the slides were deparaffinized and rehydrated, followed by antigen retrieval and the elimination of endogenous peroxidase activity. Subsequently, the slides underwent blocking with 3% BSA before being incubated with primary antibodies against TMBIM1, YBX1, CCL2, PD-L1, CD8, and CD33, at dilutions between 1:1000 and 1:100. IHC Score: IHC scoring was performed using a semi-quantitative system combining the intensity of staining and the percentage of positive cells. IHC scoring was performed using a semi-quantitative system combining the intensity of staining and the percentage of positive cells. The staining levels were assessed by multiplying the positivity (0: none of positive cell; 1: positive cell rate less than 10 %; 2: positive cell rate between 11 % and 50 %; 3: positive cell rate between 51 % and 80 %; 4: positive cell rate exceed 80 %) and intensity scores (0: no coloration; 1: pale yellow; 2: yellow; and 3: clay bank) 25. Based on the acquired scores, the classification for staining levels is as follows: Negative (score = 0, -), weakly positive (score = 1 to 4, +), moderately positive (score = 6 to 9, ++), and strongly positive (score>9, +++). Next, we categorized the patients into two groups based on TMBIM1 expression levels: one with low expression (-/+, score<6) and the other with high expression (++/+++, score≥6), and subsequently conducted survival analyses.

### Immunofluorescence staining

Cells were fixed and permeabilized, then treated with a blocking solution containing 5% BSA before being incubated with primary antibodies: Flag (1:500; Sigma, F1802, USA) and YBX1 (1:50; Santa Cruz; sc-101198, USA). Following this, the samples were incubated with secondary antibodies, specifically Alexa Fluor® 488 (1:1000; Cell Signaling Technology; 8877, USA) and DyLight™ 594 Phalloidin (1:1000; Cell Signaling Technology; 12877, USA). To visualize the nuclei, SlowFade™ Glass Soft-set Antifade Mountant containing DAPI (Invitrogen, USA) was applied.

### Chemotaxis assays

Purified MDSCs (2×10⁴ cells) isolated from PBMCs of healthy donors were placed in the upper chamber of a transwell system, while cell culture supernatants were added to the lower chamber. Cell culture supernatants and recombinant CCL2 were treated with 5 μg/mL anti-CCL2 antibody before addition to MDSCs and CD8^+^ T cells to inhibit the stimulatory effects of CCL2. The cells were incubated for 24 hours to allow migration. Following incubation, the number of migrated cells in the lower chamber was quantified using flow cytometry. The migration index (chemotaxis index) was determined as the ratio of migrated cells in response to the tested supernatant to those migrating in response to the control medium (migration index = number of migrated cells/tested supernatant ÷ number of migrated cells/control medium). Each experiment was performed independently in triplicate. A comprehensive list of reagents and antibodies is provided in Table S3.

### Mouse xenograft models and *in vivo* treatments

Six-week-old female nude mice were sourced from Shanghai SLAC Laboratory and housed in a specific pathogen-free environment in accordance with institutional guidelines. The mice were randomly assigned to two or four subgroups, with five mice per group. To establish subcutaneous tumor xenograft models, PANC-1 cells were inoculated subcutaneously into the left flanks of the mice. Once palpable tumors formed, we monitored their size biweekly, calculating tumor volume using the formula: length × width² × 0.5. After euthanizing the mice with CO_2_, tumor specimens were surgically excised. These specimens were either digested for flow cytometry analysis or fixed in paraformaldehyde for subsequent IHC staining.

For the establishment of orthotopic tumor allograft models, Pan02 cells were orthotopically inoculated into the pancreas of wild-type C57BL/6 mice. After euthanizing the mice with CO_2_, the weight of each tumor was measured. Tumor specimens were either digested for flow cytometry analysis or fixed in paraformaldehyde for subsequent IHC staining. Additionally, C57BL/6J mice received intraperitoneal injections of a neutralizing antibody against PD-1. A comprehensive list of the neutralizing antibodies used is provided in Table S4.

### Flow cytometry analysis

Mouse tumor tissues were excised and minced, then passed through 70 µm pore size filters to obtain a single-cell suspension. Following incubation with Fc block, the cells were stained with fluorochrome-conjugated antibodies for surface marker analysis. The stained cells were then analyzed using a Flow Cytometer (BD FACSCanto II or BD LSRFortessa, USA). FlowJo software was utilized for data analysis. A list of the antibodies used in the flow cytometry experiments can be found in Table S3.

### Kaplan-Meier survival analysis

Patients were divided into two groups based on TMBIM1 expression: low expression (score < 6) and high expression (score ≥ 6), allowing for subsequent survival analyses. The survival periods were illustrated using Kaplan-Meier curves, and the log-rank test was employed to compare the survival outcomes between the groups. A p-value of less than 0.05 was deemed statistically significant (P < 0.05).

### Statistical analysis

All experimental data were analyzed using GraphPad Prism 10. Data were represented as mean ± standard deviation (SD). For comparisons between two groups, an unpaired two-tailed Student's t-test was used. For comparisons among multiple groups, one-way ANOVA followed by Tukey's multiple comparisons test was employed. A significance threshold of P < 0.05 was established to denote statistically significant differences. Statistical significance was indicated as follows: *P < 0.05, **P < 0.01, **P < 0.001. All experiments were performed in triplicate (n = 3) unless otherwise stated.

## Results

### TMBIM1 is highly expressed in pancreatic cancer tissues and cell lines and promotes pancreatic cancer cell proliferation and migration

**Figure 1:**
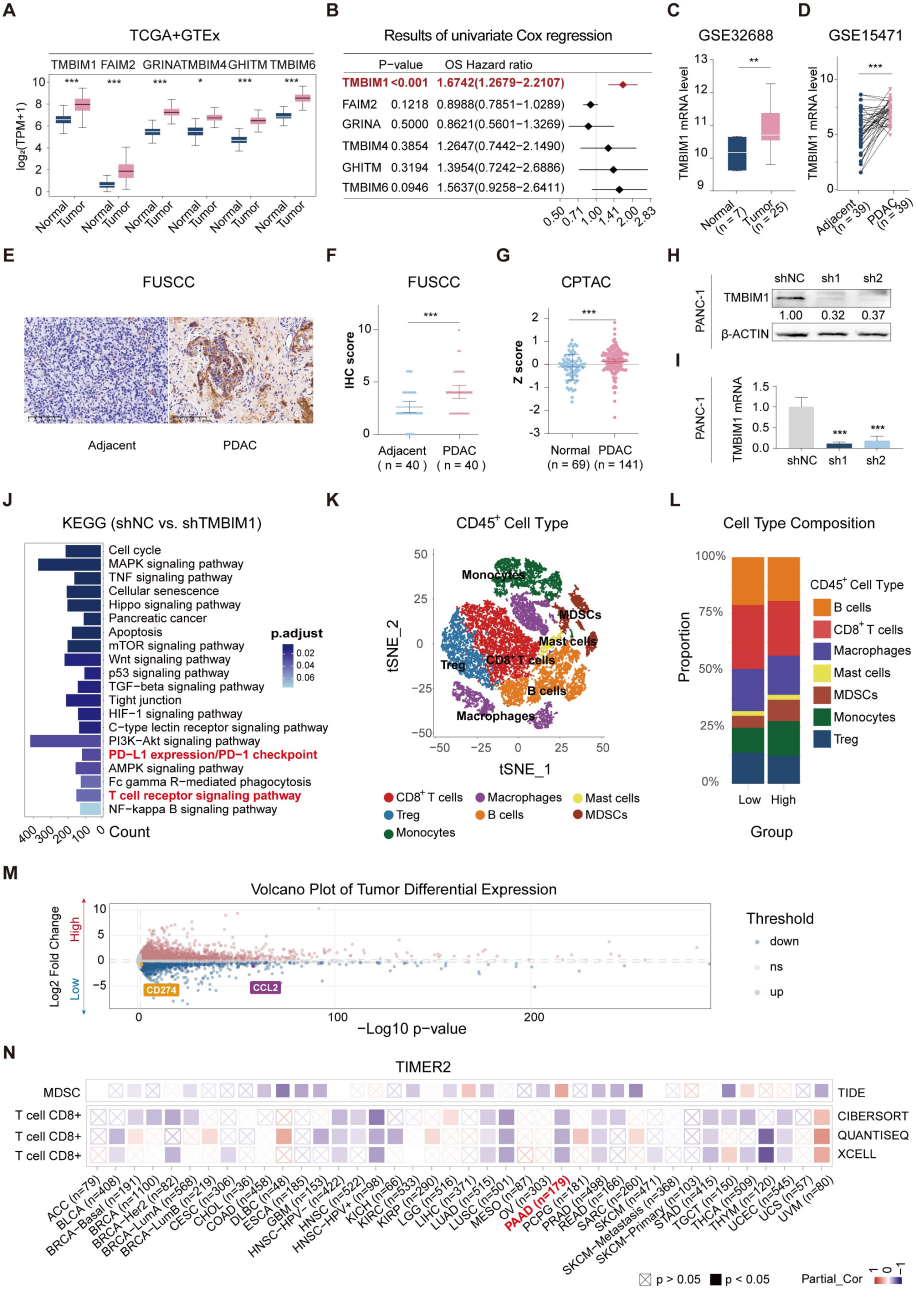
** TMBIM1 expression and its impact on pancreatic cancer progression and immune microenvironment. (A)** Boxplot showing the relative mRNA expression levels of the TMBIM family members (TMBIM1, FAIM2, GRINA, TMBIM4, GHITM, and TMBIM6) in normal and PDAC tissues from the TCGA and GTEx datasets. Statistical significance was determined using the Wilcoxon rank-sum test. **(B)** Forest plot displaying the overall survival (OS) hazard ratio (HR) of TMBIM family members in PDAC, with TMBIM1 exhibiting a significant correlation with poor prognosis (P < 0.001). (C, D) TMBIM1 mRNA expression in normal and tumor tissues analyzed in the GSE32688 dataset **(C)** and paired adjacent and PDAC tissues in the GSE15471 dataset **(D)**. Statistical significance was determined using Student's t test **(C)** and paired t test **(D)**. (E-F) IHC analysis of TMBIM1 protein expression in adjacent and PDAC tissues. Representative IHC images (left) and IHC scores from the FUSCC cohort (n = 40) (right). Scale bars = 100 μm. Statistical analysis was performed using paired t tests. **(G)** TMBIM1 protein expression (z-scores) comparison between normal and tumor tissues from the CPTAC dataset (< 0.001). (H-I) Validation of TMBIM1 knockdown efficiency in PANC-1 cells using shRNA constructs (shTMBIM1#1 and shTMBIM1#2) at the protein **(H)** and mRNA **(I)** levels. The data are presented as the mean ± standard deviation (SD). Statistical significance was determined using Student's t test. *P < 0.05; **P < 0.01; ***P < 0.001; ns, not significant. **(J)** KEGG pathway enrichment analysis of differentially expressed genes between the shTMBIM1#1 and shNC groups in PANC-1 cells. Pathways related to immune regulation, such as PD-L1 expression, PD-1 checkpoint signaling, and T-cell receptor signaling, were significantly enriched. **(K)** t-SNE plot highlighting the distribution of CD45^+^ immune cells in the PDAC microenvironment. **(L)** Bar plot of the cell type composition in the TMBIM1 high- and low-expression groups, demonstrating a greater proportion of MDSCs and a lower proportion of CD8^+^ T cells in the TMBIM1 high-expression group. **(M)** Volcano plot of differentially expressed genes between the TMBIM1 high- and low-expression groups. Notably, PD-L1 and CCL2 were significantly downregulated in the TMBIM1-low group. **(N)** Heatmap of partial correlations between TMBIM1 expression and immune cell infiltration scores across multiple cancer types, with a focus on CD8^+^ T cells and MDSCs. TMBIM1 is positively correlated with MDSC infiltration and negatively correlated with CD8^+^ T-cell infiltration in PDAC.

To investigate the expression of the TMBIM superfamily members (TMBIM1, FAIM2, GRINA, TMBIM4, GHITM, and TMBIM6), we compared their expression levels between patient tumor tissues from The Cancer Genome Atlas-Pancreatic Adenocarcinoma (TCGA-PAAD) datasets and normal pancreatic tissues from the GTEx dataset (GEPIA2.0). All six family members presented significantly increased expression in pancreatic cancer tissues (Figure 1A). The univariate Cox analysis was performed to assess the prognostic significance of the TMBIM family for OS in patients from the TCGA-PAAD cohort, revealing that TMBIM1 had the highest hazard ratio (HR) (Figure 1B, HR = 1.6742 [1.2679-2.2107], P < 0.001). Moreover, the mRNA expression profile of TMBIM1 across various cancers is shown in Figure S1A, and its expression in pancancer cell lines is presented in Figure S1B, with the highest levels observed in pancreatic cancer cell lines. Also, TMBIM1 expression was significantly associated with poor prognosis across multiple clinical outcomes, including the disease-free interval, disease-specific survival, the progression-free interval, and OS (Figure S1C). Receiver operating characteristic (ROC) curve analysis revealed that TMBIM1 was highly effective in distinguishing pancreatic cancer tissues from normal tissues (Figure S2A), and its expression level was positively correlated with both tumor stage and grade (Figure S2B-C).

We further validated the differential expression of TMBIM1 via the Gene Expression Omnibus (GEO) datasets GSE32688 and GSE15471, confirming its elevated expression in pancreatic cancer tissues compared with normal tissues from healthy controls (Figure 1C-D). Immunohistochemical analysis of 40 PDAC patient samples and 40 adjacent normal pancreatic tissue samples from the Fudan University Shanghai Cancer Center (FUSCC) also revealed significantly increased TMBIM1 protein levels in PDAC tissues (Figure 1E-F, P < 0.001). These results align with observations from the Clinical Proteomic Tumor Analysis Consortium (CPTAC) datasets (https://proteomics.cancer.gov/programs/cptac), which revealed notably different TMBIM1 protein levels between pancreatic tumors and normal tissues (Figure 1G).

Additionally, we evaluated TMBIM1 expression at both the mRNA and protein levels across seven cell lines: HPDE, Capan-1, CFPAC-1, AsPC-1, SW1990, MiaPaCa-2, and PANC-1 (Figure S3A-B). After successfully knocking down the TMBIM1 levels in the PANC-1 and Capan-1 (Figure 1H-I, S3C-D), we observed significantly suppressed pancreatic cancer cell growth and proliferation, as demonstrated by CCK8 assays (Figure S4A, B). Conversely, TMBIM1 overexpression in CFPAC-1 cells increased cell growth (Figure S3E-F). These results were corroborated by EdU and colony formation assays performed in Capan-1, PANC-1, and CFPAC-1 cells, which further confirmed the role of TMBIM1 in promoting cell proliferation (Figure S4D-G). Additionally, TMBIM1 knockdown in Capan-1 and PANC-1 cells significantly inhibited cell migration (Figure S4H). To assess the *in vivo* relevance of these findings, we established subcutaneous xenograft tumors in nude mice via the use of stably transfected PANC-1 cells. Compared with those in the negative control group, the tumors in the TMBIM1-knockdown group exhibited markedly smaller volumes and weights (Figure S5A-C). Immunohistochemical analysis of these tumors revealed reduced expression of Ki67, a marker of cell proliferation, in the TMBIM1-knockdown group, further underscoring the role of TMBIM1 in promoting tumor growth (Figure S5D). Collectively, these findings suggest that TMBIM1 functions as a protumorigenic protein in pancreatic cancer.

### TMBIM1 promotes MDSC infiltration and facilitates immunosuppression in the pancreatic cancer microenvironment

To investigate the molecular pathways influenced by TMBIM1, RNA sequencing (RNA-seq) analysis was performed on PANC-1 cells with normal TMBIM1 expression and on cells in which TMBIM1 was knocked down. KEGG pathway enrichment analysis revealed significant enrichment of the T-cell receptor signaling pathway and the PD-L1/PD-1 checkpoint pathway (Figure 1J). For further investigation, we analyzed single-cell RNA-seq (scRNA-seq) data from 30 PDAC samples (CRA001160 and GSE212966) (Figure S6A).

**Figure 2:**
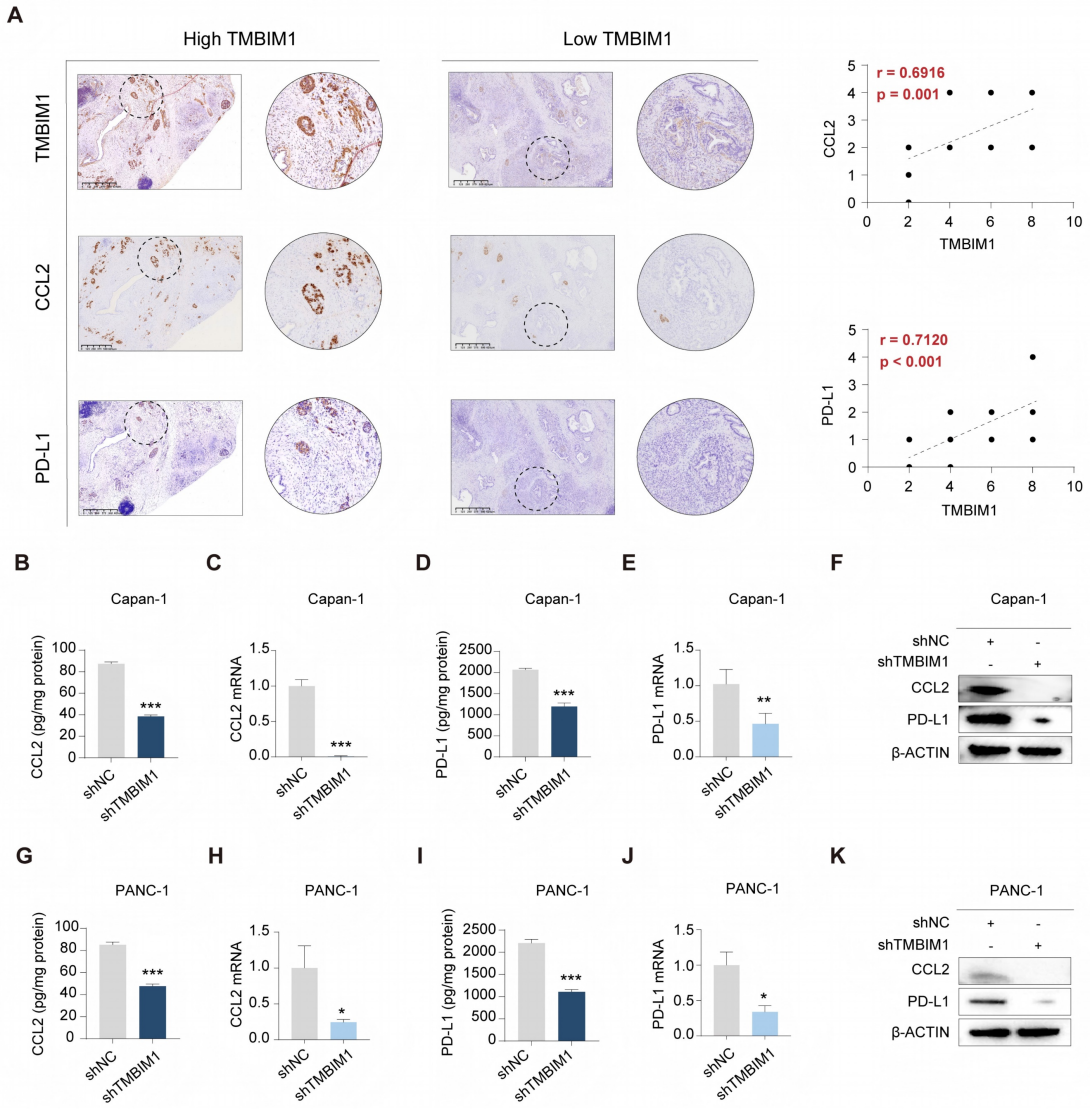
** TMBIM1 knockdown reduces CCL2 and PD-L1 expression in pancreatic cancer cells. (A)** Representative IHC images of TMBIM1, CCL2, and PD-L1 in PDAC tissues with high and low TMBIM1 expression (left panels). IHC was performed on 21 sets of paraffin-embedded PDAC tissue sections. Correlation plots show significant positive associations between TMBIM1 expression and CCL2 expression (r = 0.6916, P = 0.001) and between TMBIM1 expression and PD-L1 expression (r = 0.7120, P < 0.001) (right panels), scale bar, 625 μm. (B-E) ELISA and qPCR analyses of CCL2 (B, C) and PD-L1 (D, E) protein and mRNA levels, respectively, in Capan-1 cells following TMBIM1 knockdown (shTMBIM1) compared with the negative control (shNC). **(F)** Western blot analysis of CCL2 and PD-L1 protein levels in Capan-1 cells following TMBIM1 knockdown. (G-J) ELISA and qPCR analyses of CCL2 (G, H) and PD-L1 (I, J) protein and mRNA levels, respectively, in PANC-1 cells following shTMBIM1 compared with those following shNC. **(K)** Western blot analysis of CCL2 and PD-L1 protein levels in PANC-1 cells following TMBIM1 knockdown. β-ACTIN was used as a loading control. The data are presented as the means ± SDs. *P < 0.05; **P < 0.01; ***P < 0.001.

The scRNA-seq data were merged, normalized, and batch-corrected before being subjected to unsupervised clustering, which identified distinct cell populations within the TME. Key markers for each cell type were identified, revealing major cell populations within the pancreatic cancer microenvironment, including acinar cells, mast cells, plasma B cells, epithelial and endothelial cells, stromal cells, myeloid cells, T and B lymphocytes, and malignant cells (Figure 2S6B-C). Tumor cells were stratified into high and low TMBIM1 expression groups, resulting in the identification of 2,227 upregulated and 1,345 downregulated genes through differential gene expression analysis (Table S6). To identify critical genes within the pancreatic cancer TME, we integrated and analyzed RNA-seq and scRNA-seq data and a curated chemokine list 25. This integrative analysis, depicted in the Venn diagram (Figure S6D), highlighted key overlapping genes. Notably, CCL2 and PD-L1 emerged as significant candidates, which aligned with the RNA-seq findings (Figure 1M).

To delve deeper into immune cell infiltration, we isolated CD45^+^ cells, performed further clustering, and annotated the resulting cell types (Figure 1K, S6E). A comparative analysis between the high and low TMBIM1 expression groups revealed a significant increase in MDSC infiltration and a decrease in CD8^+^ T-cell infiltration within the high TMBIM1 group (Figure 1L). These observations were corroborated using the TIMER 2.0 platform and TCGA-PAAD data (Figure 1N). Additionally, we detected a greater abundance of PMN-MDSCs in the scRNA-seq data corresponding to high TMBIM1 expression (Figure S6F).

### TMBIM1 drives CCL2 upregulation to promote tumor malignancy and increase MDSC infiltration in pancreatic cancer

To explore the associations between the expression levels of TMBIM1 and CCL2 and PD-L1 in PDAC, immunohistochemistry (IHC) staining was carried out on tumor tissues. The analysis revealed significantly elevated CCL2 and PD-L1 expression in the high-TMBIM1 group, and strong positive correlations were revealed (r = 0.6916 and r = 0.7120, respectively) (Figure 2A). Additionally, real-time quantitative PCR (qPCR) and ELISA analyses demonstrated that TMBIM1 knockdown significantly reduced CCL2 and PD-L1 expression levels (Figure 2B-E, 2G-K). These findings were corroborated by the Western blot analyses of both Capan-1 and PANC-1 cells (Figure 2F, 2K), indicating that TMBIM1 plays a role in regulating CCL2 and PD-L1 expression in pancreatic cancer cells.

To investigate whether CCL2 acts downstream of TMBIM1 and promotes pancreatic cancer cell proliferation and migration, we utilized the Capan-1 and PANC-1 cell lines. Western blot analysis confirmed the successful overexpression of CCL2 (CCL2-OE) and the knockdown of TMBIM1 in both cell lines (Figure S7A). Cell proliferation assays, including CCK-8 and EdU incorporation assays, revealed that CCL2-OE significantly increased cell proliferation. Notably, the decreased proliferation caused by shTMBIM1 was effectively abrogated by simultaneous CCL2-OE (Figure S7B-C). Similarly, colony formation assays revealed an increase in colony numbers with CCL2-OE, whereas the opposite effects were observed when CCL2-OE was combined with TMBIM1 knockdown (Figure S7D). Furthermore, Transwell migration assays revealed that CCL2-OE increased cell migration, whereas shTMBIM1 significantly decreased it. Importantly, migratory capacity was partially restored in TMBIM1-knockdown cells upon CCL2-OE (Figure S7E). Taken together, these findings suggest that TMBIM1 promotes pancreatic cancer cell proliferation and migration through mechanisms involving CCL2.

**Figure 3:**
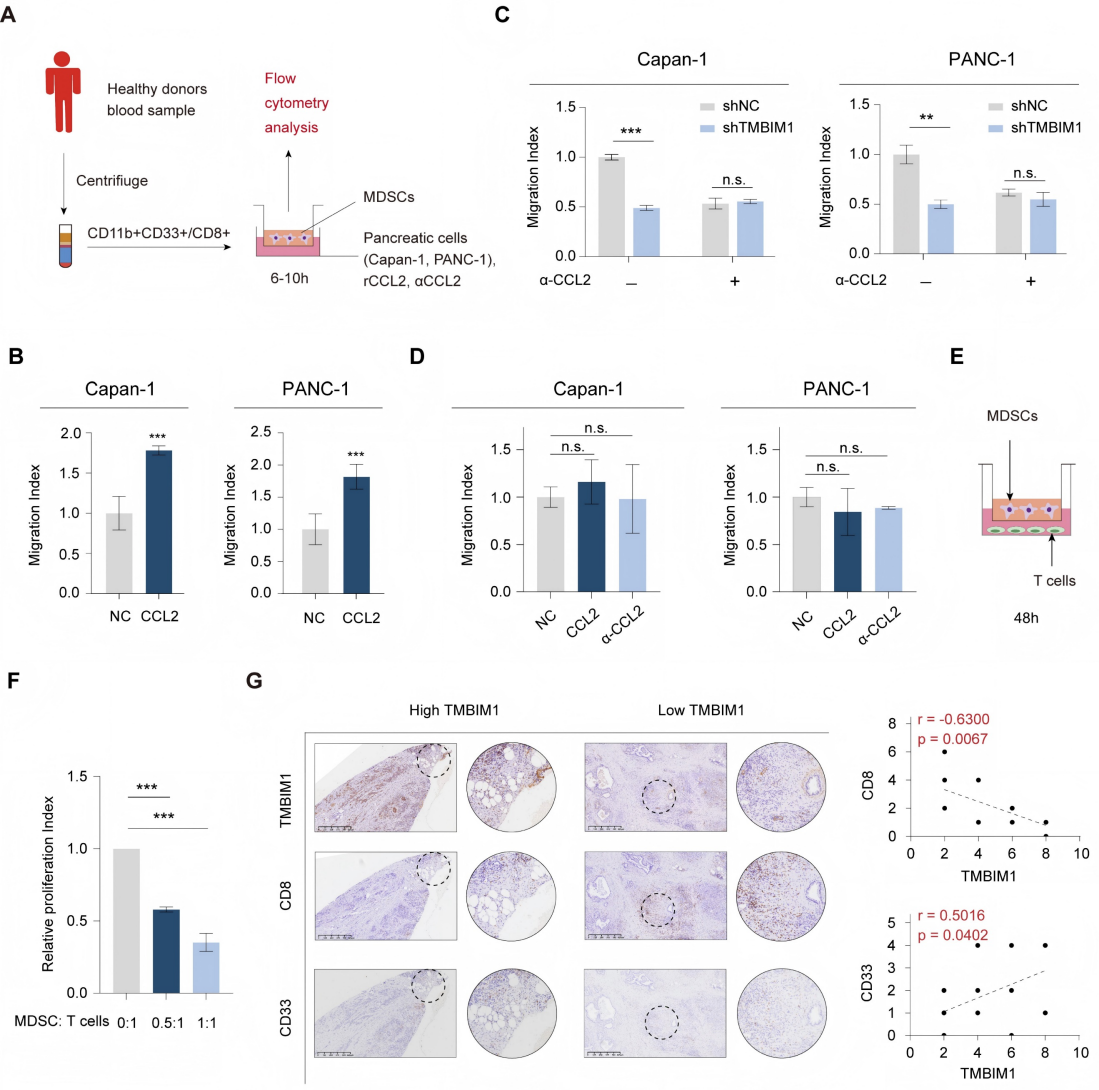
** CCL2 mediates MDSC recruitment, influencing T-cell proliferation in the pancreatic cancer microenvironment. (A)** Schematic diagram of the experimental workflow. Peripheral blood mononuclear cells (PBMCs) were isolated from healthy donors, and MDSCs (CD11b^+^CD33^+^) were sorted by flow cytometry. Human pancreatic cancer cells (Capan-1 and PANC-1) cultured with recombinant CCL2 (rCCL2), anti-CCL2 antibody (αCCL2) or control medium were cocultured with MDSCs in transwell migration assays for 6-10 hours, followed by flow cytometry analysis. **(B)** Migration index of MDSCs exposed to conditioned media from Capan-1 and PANC-1 cells with or without αCCL2. **(C)** Migration index of MDSCs in Transwell assays using conditioned media from shTMBIM1 or shNC cells with or without the αCCL2 blocking antibody. **(D)** The migration index of CD8^+^ T cells was assessed in the presence or absence of rCCL2 or αCCL2 blocking antibody in the culturing medium of Capan-1 (left) and PANC-1 (right). (E-F) MDSC-mediated T-cell suppression assay. Schematic **(E)** and relative proliferation indices **(F)** of T cells cocultured with MDSCs at different ratios (MDSC:T-cell ratios = 0:1, 0.5:1, 1:1) for 48 hours. **(F)** Migration index of Capan-1 and PANC-1 cells exposed to rCCL2 with or without an αCCL2 blocking antibody. **(G)** Representative IHC staining of TMBIM1, CD8 (T cells), and CD33 (MDSCs) in PDAC tissues with high and low TMBIM1 expression (left panels). IHC was performed on 21 sets of paraffin-embedded PDAC tissue sections. Correlation plots revealed significant negative associations between TMBIM1 expression and the proportion of CD8^+^ T cells (r = -0.6300, P = 0.0057) and significant positive associations between TMBIM1 expression and the proportion of CD33^+^ MDSCs (r = 0.5019, P = 0.0402) (right panels), scale bar, 625 μm. The data are presented as the means ± SDs. n.s., not significant; **P < 0.01; ***P < 0.001.

Notably, previous studies have demonstrated that MDSC migration relies on the interaction between the chemokine receptor CCR2 and its ligand CCL2 26, 27. Next, we assessed the role of CCL2 in driving CD8^+^ T-cell and MDSC migration *in vitro* (Figure 3A). T cells and MDSCs were isolated from human peripheral blood using flow cytometry sorting. The addition of recombinant CCL2 (rCCL2) to the supernatants of Capan-1 and PANC-1 cells in the coculture system with MDSCs significantly increased the migration of MDSCs (Figure 3B), and the results of subsequent chemotaxis assays indicated that compared with culture medium from shTMBIM1 cells, culture medium from Capan-1 and PANC-1 cells increased the migration of MDSCs (Figure 3C). Next, in coculture experiments of CD8^+^ T cells, the migration index remained unaffected by the presence of recombinant CCL2 (rCCL2) or an anti-CCL2 antibody (αCCL2) (Figure 3D). Additionally, we confirmed the immunosuppressive effects of the MDSCs through a T-cell proliferation assay (Figure 3E-F).

Additionally, as shown in Figure S8A and 8B, we further examined the impact of Capan-1 and PANC-1 cells (shNC, shTMBIM1, and shTMBIM1+rCCL2 groups) on the chemotaxis of T cells. The results revealed no statistically significant differences between the two groups, and the addition of rCCL2 did not influence the infiltration of CD8^+^ T cells. This finding is predictable because CD8^+^ T cells lack CCR2 on their surface. Hence, the absence of a chemotactic response aligns with our initial hypothesis. In line with this observation, single-cell transcriptomic analysis suggested that the differences in CD8^+^ T-cell composition between the TMBIM1 high- and low-expression groups are likely attributed to the differential infiltration of MDSCs. The immunosuppressive effect of MDSCs may indirectly inhibit CD8^+^ T-cell proliferation.

To further explore the association between TMBIM1 expression and immune cell infiltration in PDAC, IHC was conducted on tumor biopsies to assess MDSC and CD8^+^ T-cell infiltration. The IHC results revealed a negative correlation between TMBIM1 expression and CD8^+^ T-cell infiltration, whereas a positive correlation was observed with MDSC infiltration (Figure 3G). These findings suggest that CCL2 may play a crucial role in the chemotaxis of MDSCs in PDAC. Overall, these results imply that TMBIM1 significantly regulates the establishment of an immunosuppressive microenvironment in PDAC by influencing MDSC infiltration.

### TMBIM1 induces YBX1 protein phosphorylation and translocation into the nucleus

**Figure 4:**
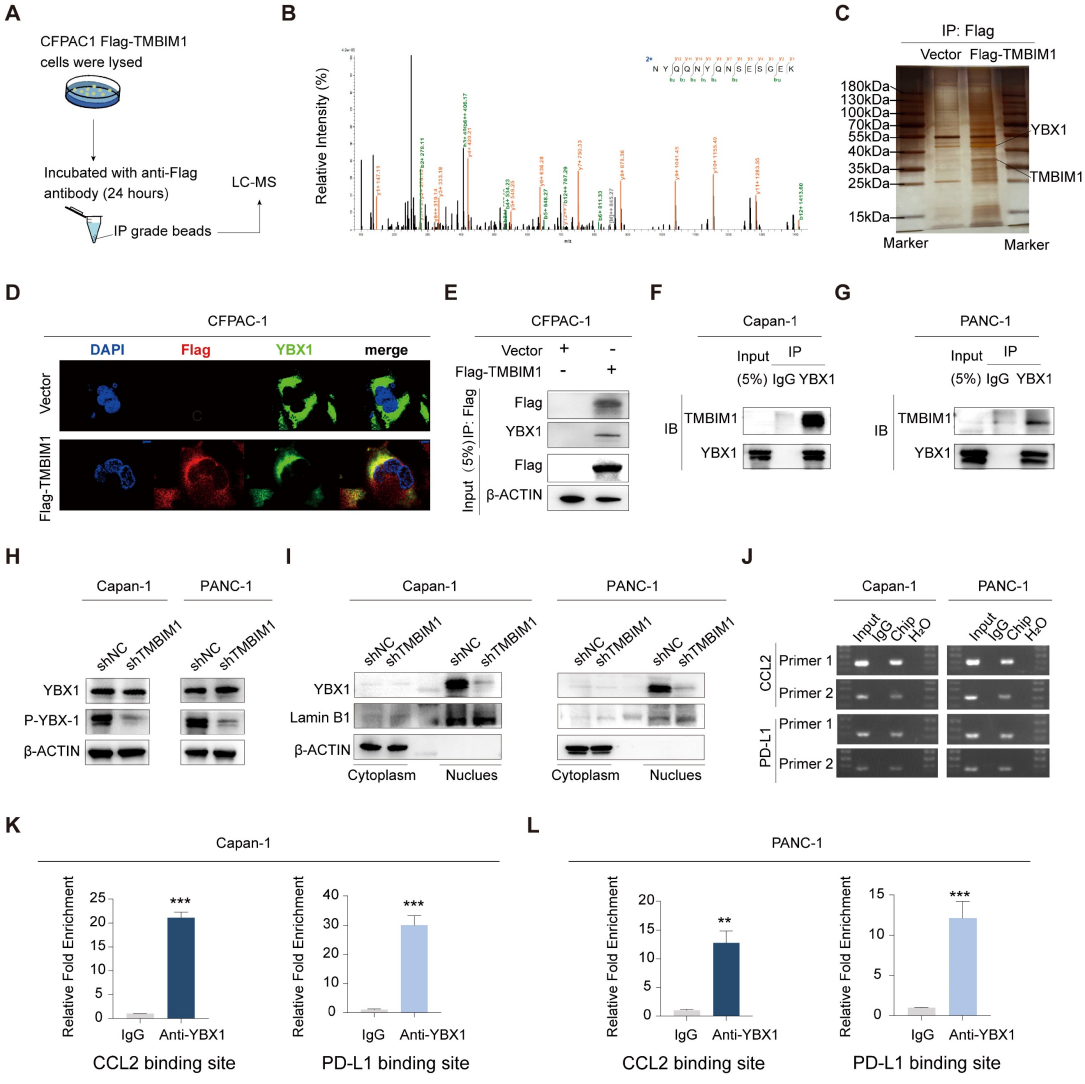
** TMBIM1 interacts with YBX1 and regulates CCL2 and PD-L1 transcription in pancreatic cancer cells. (A)** Schematic illustration of the IP‒MS experiment used to identify TMBIM1-interacting proteins. CFPAC-1 cells expressing Flag-tagged TMBIM1 were lysed and subjected to IP with an anti-Flag antibody, followed by LC‒MS analysis. **(B)** Mass spectrometry analysis showing the relative intensity of proteins interacting with Flag-TMBIM1. YBX1 was identified as a significant interacting partner. **(C)** IP followed by immunoblotting confirming the interaction between Flag-TMBIM1 and YBX1 in CFPAC-1 cells. The vector control was used as a negative control. **(D)** Immunofluorescence images of CFPAC-1 cells expressing either vector or Flag-TMBIM1, showing colocalization of TMBIM1 (Flag) and YBX1. DAPI was used to stain the nuclei. **(E)** Co-IP assays in CFPAC-1 cells further validated the interaction between Flag-TMBIM1 and YBX1 by IP with Flag and immunoblotting for YBX1. (F, G) Co-IP assays in Capan-1 and PANC-1 cells showing the endogenous interaction between TMBIM1 and YBX1. **(H)** Western blot analysis of YBX1 and P-YBX1 (S102) expression levels in Capan-1 and PANC-1 cells upon TMBIM1 knockdown, revealing decreased phosphorylation of YBX1. **(I)** Western blot analysis of YBX1 in the cytoplasmic and nuclear fractions of Capan-1 and PANC-1 cells upon TMBIM1 knockdown. Lamin B1 was used as a nuclear marker, and β-actin was used as a cytoplasmic marker. **(J)** ChIP assays showing YBX1 binding at the CCL2 and PD-L1 promoter regions in Capan-1 and PANC-1 cells. IgG was used as a control. (K, L) Quantitative ChIP analysis indicating significant enrichment of YBX1 at the CCL2 and PD-L1 binding sites in Capan-1 and PANC-1 cells compared with the IgG control.

To investigate the proteins that interact with TMBIM1, we performed immunoprecipitation (IP) in CFPAC-1 cells stably overexpressing Flag-TMBIM1, followed by liquid chromatography-mass spectrometry (LC‒MS) analysis (Figure 4A). YBX1, a prominent transcription factor, is recognized for its ability to undergo phosphorylation and translocate into the nucleus, where it induces the transcription of PD-L1 (25). Notably, our LC‒MS analysis revealed a significant interaction between TMBIM1 and YBX1 (Figure 4B). We also performed molecular docking analysis using http://hdock.phys.hust.edu.cn/ to further confirm the binding relationship between TMBIM1 and YBX1. The results, as summarized in Table S5, revealed that specific amino acid residues in TMBIM1 form stable hydrogen bonds with YBX1. Notably, residues THR-7, ARG-282, ARG-279, ARG-247, ARG-253, TYR-241, and TYR-238 of YBX1 were predicted to be crucial binding sites for TMBIM1 (Figure S9). Further validation of the TMBIM1-YBX1 interaction was achieved through silver staining following Western blot analysis (Figure 4C). Furthermore, immunofluorescence staining revealed marked colocalization of TMBIM1 and YBX1 in CFPAC-1 cells, with significant overlap in the cytoplasmic compartments (Figure 4D). To investigate whether this interaction occurs in other pancreatic cancer cell lines, we performed immunoprecipitation assays on Capan-1 and PANC-1 cells using an anti-YBX1 antibody. These assays provided additional evidence for the TMBIM1-YBX1 interaction (Figure 4E-G).

These findings confirm that TMBIM1 indeed increases the transcriptional activity of YBX1. Notably, the knockdown of TMBIM1 resulted in decreased phosphorylation of YBX1 (S102) without affecting its overall expression level (Figure 4H). These findings suggest a potential alteration in the distribution of YBX1 between the nucleus and cytoplasm. To test this hypothesis rigorously, we employed a nuclear-cytosolic extraction kit for protein separation. Our analysis revealed a reduction in intranuclear YBX1 levels and an increase in extranuclear YBX1 levels following TMBIM1 downregulation, indicating that TMBIM1 is crucial for facilitating the nuclear entry of YBX1 (Figure 4I), which aligns with our expectations. On the basis of these results, we established that TMBIM1 modulates tumor CCL2 and PD-L1 expression through the regulation of YBX1.

### TMBIM1 and YBX1 collaborate to control CCL2 and PD-L1 transcription in PDAC

**Figure 5:**
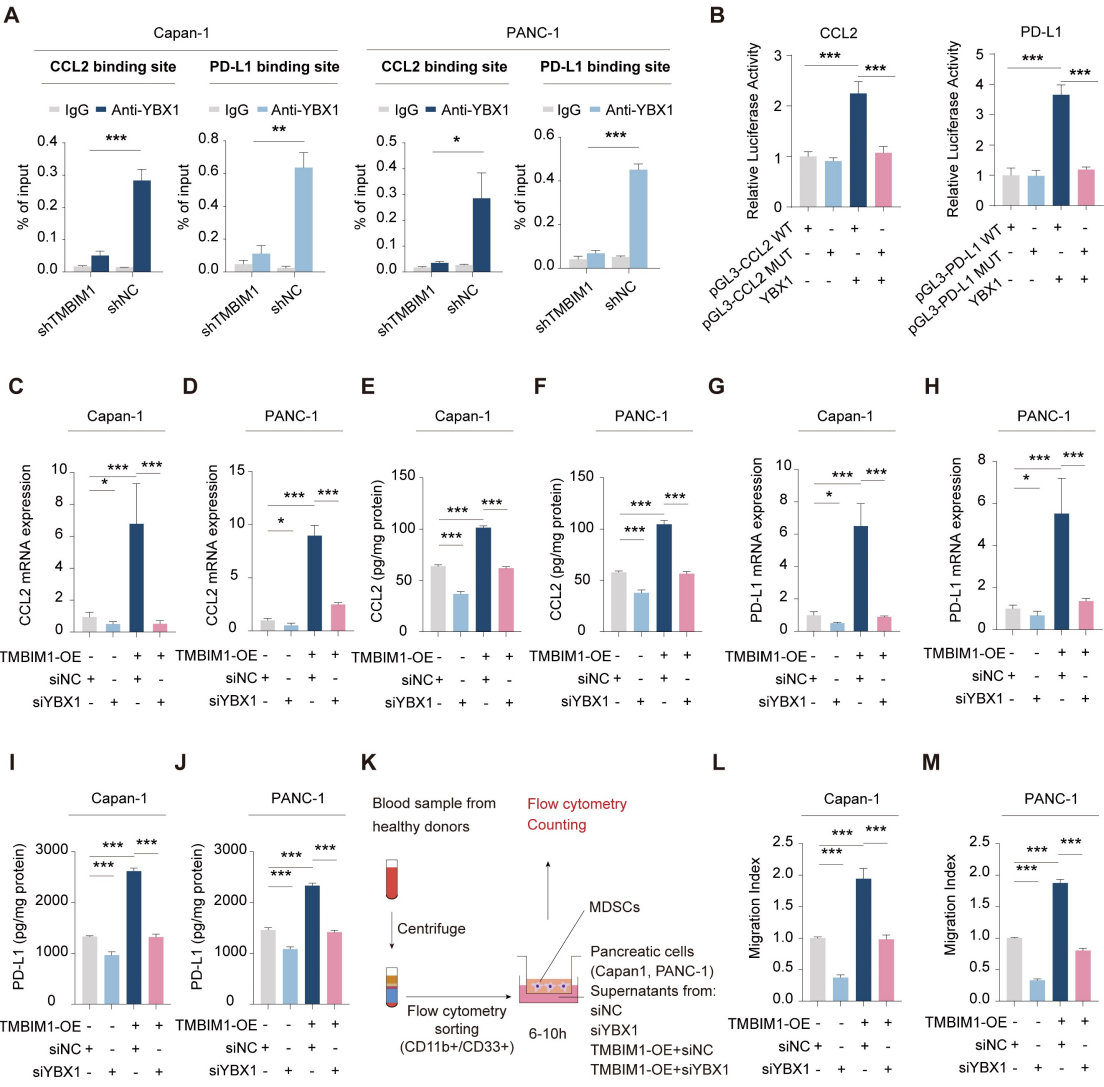
** TMBIM1 regulates CCL2 and PD-L1 expression via YBX1 binding in pancreatic cancer cells.** ChIP-qPCR analysis in Capan-1 and PANC-1 cells demonstrates that YBX1 enrichment at the binding site of the promoters of CCL2 and PD-L1 is significantly higher in shNC compared to shTMBIM1 cells. **(B)** Luciferase reporter assays in Capan-1 cells transfected with either WT or MUT constructs of the CCL2 (left) or PD-L1 (right) promoter, with or without YBX1 overexpression. Relative luciferase activity was measured to assess promoter activity. (C-D) qPCR analysis of CCL2 mRNA expression in **(C)** Capan-1 and **(D)** PANC-1 cells overexpressing TMBIM1 with or without siYBX1 (n=3). (E-F) ELISA to measure the CCL2 protein levels in the supernatants of Capan-1 **(E)** and PANC-1 **(F)** cells under the same conditions. (G-H) qPCR analysis of PD-L1 mRNA expression in Capan-1 **(G)** and PANC-1 **(H)** cells treated with siNC, siYBX1, TMBIM1-OE+siNC, or TMBIM1-OE+siYBX1. (I, J) ELISA to measure PD-L1 protein levels in the supernatants of Capan-1 **(I)** and PANC-1 **(J)** cells under the same conditions. **(K)** Schematic of the MDSC migration assay. MDSCs were isolated from healthy donor blood samples using flow cytometry sorting (CD11b^+^CD33^+^), followed by coculture with supernatants from siNC, siYBX1, TMBIM1-OE+siNC, or TMBIM1-OE+siYBX1-treated Capan-1 or PANC-1 cells. (L, M) Transwell migration assay quantifying MDSC migration toward conditioned medium from Capan-1 **(L)** or PANC-1 **(M)** cells. Migration indices were calculated for each condition (n=3). The data are presented as the means ± SDs. n.s., not significant; **P < 0.01; ***P < 0.001.

Considering the colocalization and functional roles of TMBIM1 and YBX1 in the Capan-1 and PANC-1 cell lines, we propose that nuclear YBX1 may interact with the promoter regions of CCL2 and PD-L1. To investigate this, we designed 10 pairs of primers targeting all potential binding sites within these promoter regions and performed chromatin immunoprecipitation (ChIP) assays to identify the binding sites for TMBIM1. ChIP assays revealed that YBX1 interacts with the binding sites in the promoters of CCL2 and PD-L1 (Figure 4J). We subsequently conducted ChIP‒qPCR assays to evaluate the interactions of TMBIM1 and YBX1 with chromatin elements in the promoter regions of CCL2 and PD-L1 (Figure 4K-L). The results indicated a significant decrease in YBX1 occupancy at the promoter regions following TMBIM1 knockdown (Figure 5A).

To identify the specific binding elements within the promoters of CCL2 and PD-L1, we created mutant promoter constructs by altering the binding sites on the basis of the results above. The motif sequence for YBX1 was retrieved from the JASPAR database (Figure S10). To evaluate the transcriptional regulation of CCL2 and PD-L1 by YBX1, we performed luciferase reporter assays using both wild-type (WT) and mutant (MUT) CCL2 and PD-L1 promoter constructs. Our findings revealed YBX1 binding motifs within the CCL2 promoter, and mutations at these sites resulted in a marked decrease in YBX1-mediated luciferase activity (Figure 5B, left). Similarly, YBX1 was shown to be crucial for PD-L1 promoter activity, as evidenced by a significant decrease in luciferase activity when the YBX1 motif was mutated (Figure 5B, right).

qPCR and ELISA analyses demonstrated that the overexpression of TMBIM1 in both Capan-1 and PANC-1 cells resulted in substantial increases in the mRNA and protein levels of CCL2 and PD-L1 (Figure 5C-J). In contrast, the knockdown of YBX1 (siYBX1) completely abolished these effects, indicating that the TMBIM1-induced expression of CCL2 and PD-L1 is mediated through YBX1. Given the role of CCL2 in recruiting MDSCs, we further investigated the influence of TMBIM1 on MDSC infiltration. As shown in Figure 5K, we isolated CD11B^+^CD33^+^ MDSCs from the blood of healthy donors and cocultured them with conditioned media from Capan-1 and PANC-1 cells treated with TMBIM1-OE, siNC, or siYBX1. Flow cytometry analysis revealed that the conditioned media from TMBIM1-OE cells significantly increased MDSC recruitment, an effect that was abrogated upon YBX1 knockdown (Figure 5L-M). These findings suggest that TMBIM1 increases MDSC infiltration through YBX1-dependent mechanisms.

Taken together, these results indicate that TMBIM1 increases YBX1 activation and its translocation into the nucleus, resulting in elevated expression of CCL2 and PD-L1. This increase subsequently promotes MDSC infiltration within the TME, ultimately assisting in immune evasion.

### TMBIM1 facilitates *in vivo* tumor growth and shapes an immunosuppressive TME

**Figure 6:**
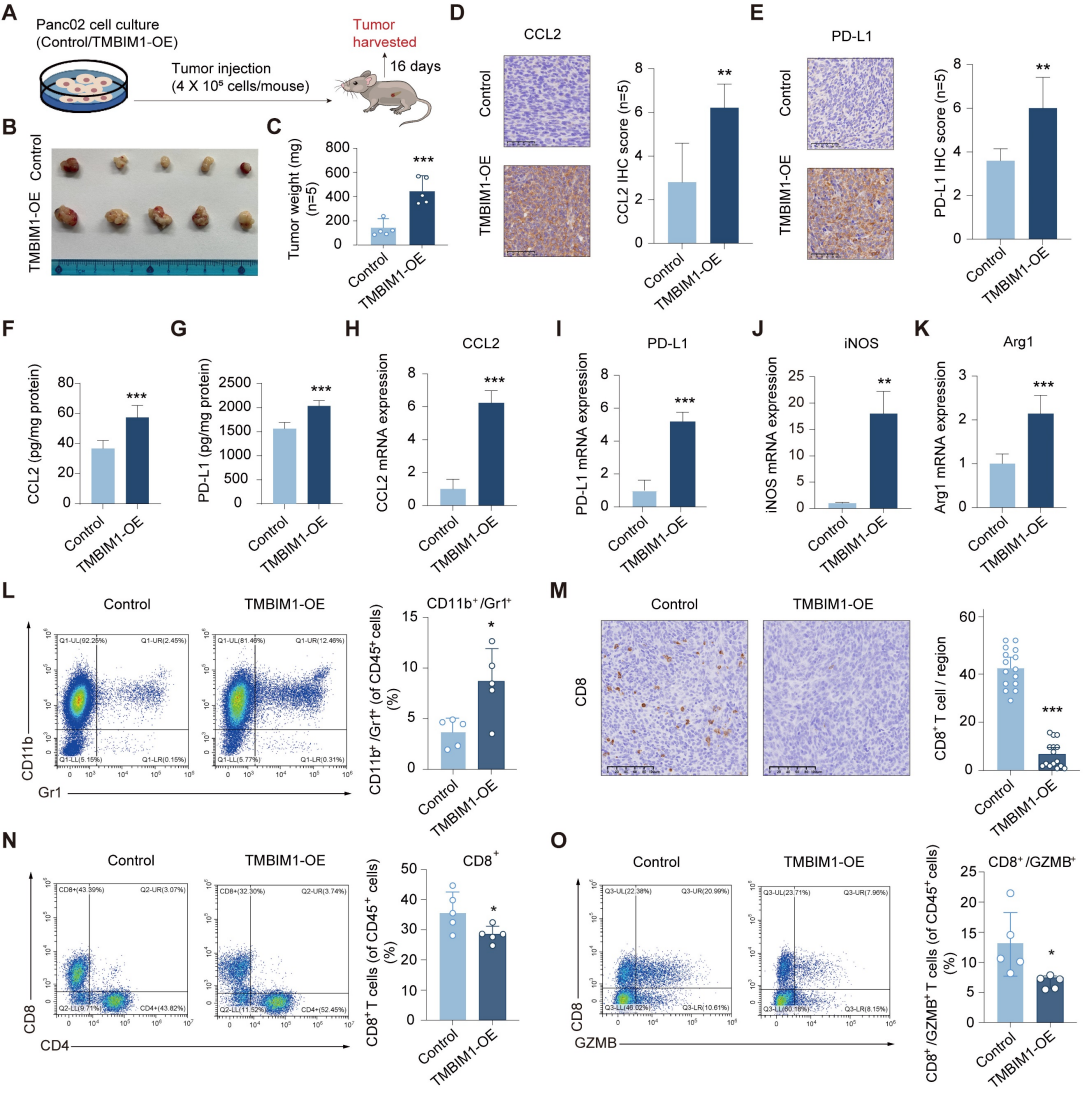
** TMBIM1 overexpression promotes tumor growth and modulates immune cell populations in murine pancreatic cancer models. (A)** Schematic representation of the experimental design. Pan02 cells with either control vector or TMBIM1-OE were injected into C57BL/6J mice. Tumors were harvested 16 days post-injection for further analysis. **(B)** Representative images of tumors excised from mice in the control and TMBIM1-OE groups (n=5). **(C)** Quantification of tumor weights (n=5). (D-E) IHC staining of tumor sections showing increased expression of CCL2 **(D)** and PD-L1 **(E)** in TMBIM1-OE tumors. Quantification of IHC staining intensity is shown on the right (D-E) (Scale bar, 100 µm; ***P < 0.001; **P < 0.01). (F-G) Levels of chemokines CCL2 **(F)** and PD-L1 **(G)** in tumor tissue lysates, as quantified by ELISA, were significantly higher in the TMBIM1-OE group compared to controls. (H-K) qPCR analysis of gene expression in tumor tissues. mRNA levels of CCL2 **(H)**, PD-L1 **(I)**, iNOS **(J)**, and Arg1 **(K)** were significantly elevated in TMBIM1-OE tumors compared to controls. **(L)** Flow cytometry analysis of CD11b^+^/Gr1^+^ MDSCs in tumor tissues. **(M)** IHC staining for CD8^+^ T cells in tumor sections showed a reduction in CD8^+^ T cell infiltration in TMBIM1-OE tumors compared to controls (Scale bar, 100 µm; *P < 0.05). (N, O) Flow cytometry analysis of CD8^+^ T cells **(N)** and CD8^+^/GZMB^+^ cytotoxic T cells **(O)** in tumor tissues. The data are presented as the means ± SDs. n.s., not significant; **P < 0.01; ***P < 0.001.

To investigate the role of TMBIM1 in tumor progression *in vivo*, we overexpressed TMBIM1 in the mouse PDAC cell line Pan02 (Figure S11). We subsequently established an orthotopic allograft tumor model using Pan02 cells in C57BL/6 mice (Figure 6A). The results revealed that tumors from the TMBIM1-OE group had a significantly greater tumor burden than those from the control group did (Figure 6B, C). Analysis of the isolated and homogenized tumor samples revealed that the intratumoral levels of CCL2 and PD-L1 were notably elevated in the TMBIM1-OE tumors (Figure 6D-I). Additionally, we observed increased mRNA expression of Arg1 and iNOS, both of which are markers associated with MDSC infiltration (Figure 6J, K). The depletion of L-arginine by iNOS and the production of Arg1 by MDSCs contribute to T-cell suppression 28.

Flow cytometry analysis of immune cells infiltrating the tumors revealed a significant increase in the CD11b^+^Gr1^+^ population, indicating a greater presence of MDSCs in the tumors overexpressing TMBIM1 (Figure 6L; see the flow cytometry gating strategy in Figure S12). Conversely, both immunohistochemical staining and flow cytometry analyses revealed a marked reduction in the number of CD8^+^ T cells within TMBIM1-OE tumors (Figure 6M, N; Figure S13). Moreover, flow cytometry confirmed a significant decrease in activated CD8^+^ T cells (CD8^+^/GZMB^+^) in tumors with TMBIM1 overexpression (Figure 6O).

### TMBIM1 downregulation increases the sensitivity of PDAC to anti-PD-1 therapy in tumor-bearing mice

**Figure 7:**
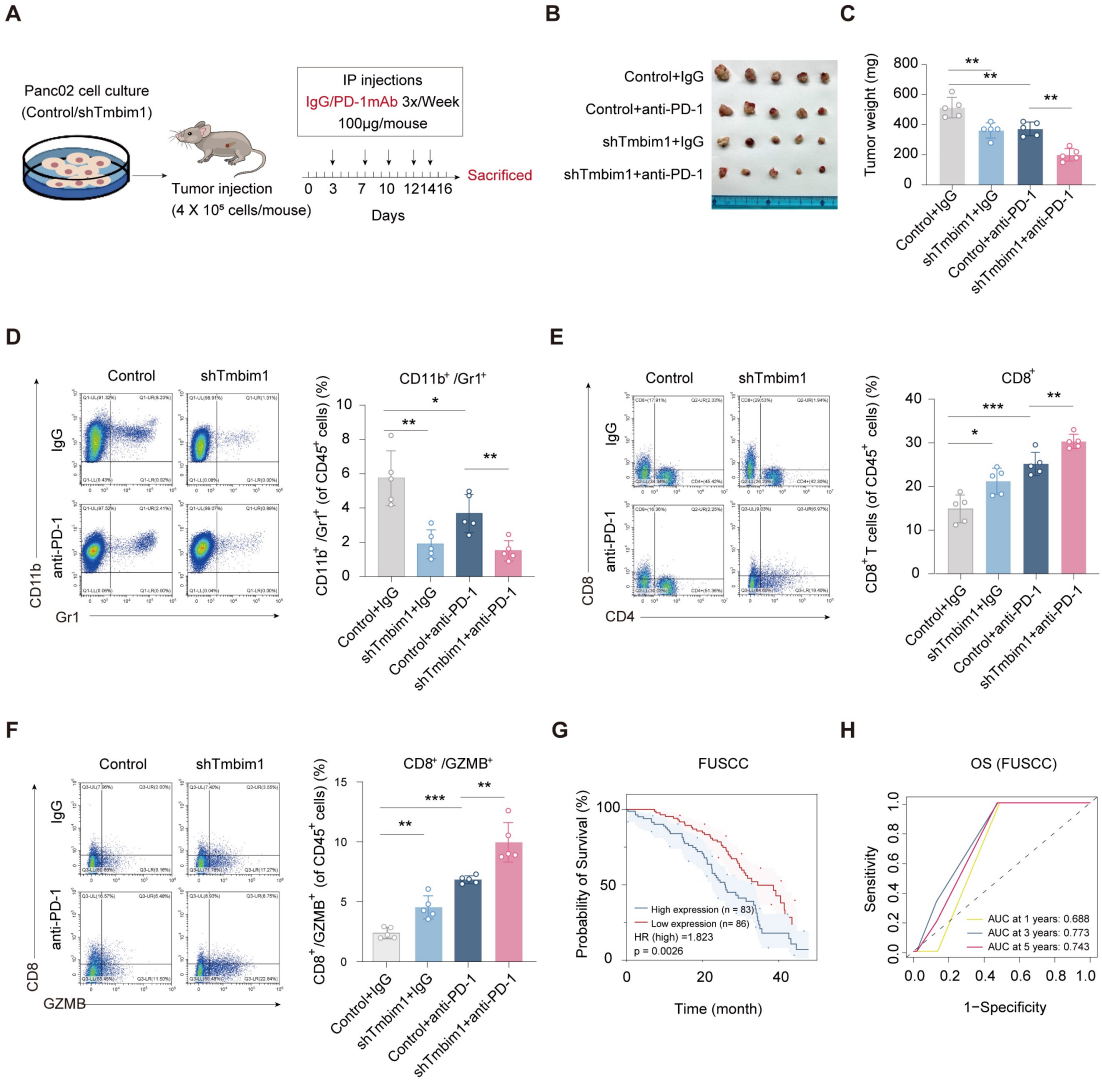
** TMBIM1 knockdown sensitizes tumor-bearing mice to anti-PD-1 blockade therapy. (A)** Schematic representation of the *in vivo* experimental setup. Pan02 cells with either control or TMBIM1 knockdown were injected into the mice, followed by intraperitoneal administration of IgG or anti-PD-1 antibody (100 µg/mouse) three times per week. Tumors were harvested on day 16 for analysis. **(B)** Representative images of tumors from the four groups: Control+IgG, Control+anti-PD-1, shTMBIM1+IgG, and shTMBIM1+anti-PD-1. **(C)** Quantification of tumor weights. Compared with those in the other groups, the tumors in the shTMBIM1+anti-PD-1 group were significantly smaller. **(D)** Flow cytometry analysis of CD11b^+^/Gr1^+^ MDSCs in tumor tissues. **(E)** Flow cytometry analysis of CD8^+^ T cells in tumor tissues. Compared with those in the other groups, the CD8^+^ T-cell infiltration in the shTMBIM1+anti-PD-1 group was significantly greater. **(F)** Flow cytometry analysis of CD8^+^/GZMB^+^ cytotoxic T cells in tumor tissues. Compared with the control treatment, the combination of TMBIM1 knockdown and anti-PD-1 therapy significantly increased the percentage of CD8^+^/GZMB^+^ cells. The data are presented as the means ± SDs. n.s., not significant; **P < 0.01; ***P < 0.001. **(G)** Kaplan‒Meier curves of OS in patients with pancreatic cancer from the FUSCC cohort (n=169). **(H)** ROC curve analysis for OS in FUSCC cohort, demonstrating prognostic accuracy of TMBIM1 at 1, 3, and 5 years.

To assess the role of TMBIM1 in immune evasion and its effect on the response to immunotherapy, we created a mouse model of *in situ* pancreatic tumors using Pan02 cells transfected with either control shRNA or shTMBIM1. The mice were randomly divided into groups and administered intraperitoneal injections of either IgG or anti-PD-1 antibodies three times per week, as illustrated in Figure 7A. After a 15-day treatment period, the tumors were excised for analysis. The tumor growth data demonstrated that the combination of TMBIM1 knockdown and anti-PD-1 treatment led to significantly smaller tumors than either treatment alone or the control (Figure 7B). Moreover, quantification of tumor weight indicated that the shTMBIM1 + anti-PD-1 group displayed the most substantial tumor suppression (Figure 7C). These findings suggest that TMBIM1 expression may contribute to resistance to anti-PD-1 therapy and that its inhibition can increase the sensitivity of pancreatic tumors to ICB therapy. Additionally, analysis of the TCIA database (https://tcia.at/home) predicted that TMBIM1 plays a role in resistance to anti-PD-1 treatment. The data indicate that the rate of nonresponse to both anti-CTLA-4 and anti-PD-1 antibodies is significantly greater in the group with high TMBIM1 expression (Figure S14).

Furthermore, we investigated the immune cell composition within the TME via flow cytometry. Notably, we observed a significant decrease in the number of CD11B^+^Gr1^+^ MDSCs in TMBIM1-knockdown tumors compared with control tumors (Figure 7D). These findings suggest that TMBIM1 promotes the recruitment of MDSCs, which are key mediators of immune suppression in pancreatic cancer. Furthermore, we examined the infiltration and activation of CD8^+^ T cells. TMBIM1 knockdown led to a notable increase in CD8^+^ T-cell populations within the tumors, particularly those expressing GZMB, a cytolytic effector molecule (Figure 7E, 7F). Importantly, this enhancement was further pronounced when TMBIM1 knockdown was coupled with PD-1 blockade, underscoring the role of TMBIM1 in constraining T-cell-mediated immune responses in pancreatic cancer.

To evaluate the clinical significance of our findings, we analyzed the relationship between TMBIM1 expression and patient prognosis across multiple datasets. Kaplan-Meier survival analyses revealed that elevated TMBIM1 expression was significantly associated with shorter OS among pancreatic cancer patients (Figure 7G; Figure S15A). Similarly, in the TCGA-PAAD cohort, TMBIM1 expression displayed a robust association with shorter OS, PFS, and DSS (Figure S15B-E). ROC curve analysis further highlighted the predictive capability of TMBIM1 in TCGA-PAAD, showing AUC values of 0.598, 0.685, and 0.725 for 1-, 3-, and 5-year OS predictions, respectively (Figure S15F). Consistently, similar trends were observed in the GSE79668 and CPTAC datasets, where elevated TMBIM1 expression was strongly associated with reduced OS probability (Figure S15G-H). Furthermore, Table 1 summarizes the outcomes of univariate and multivariate Cox regression analyses for OS in PDAC patients from the FUSCC. In the univariate analysis, factors significantly associated with poor survival included the presence of vascular cancer emboli (P = 0.021), lymph node metastasis (P = 0.038), elevated preoperative CA19-9 levels (P = 0.005), large tumor size (≥3 cm, P = 0.002), advanced T stage (P = 0.005), and high IHC scores (P = 0.003). In the multivariate cox analysis, only advanced T stage (P = 0.025) and high TMBIM1 IHC scores (P = 0.003) remained significant, confirming their status as independent prognostic factors for OS. These results suggest that TMBIM1 acts as a negative prognostic marker and may contribute to unfavorable patient outcomes by promoting immune evasion.

**Figure 8:**
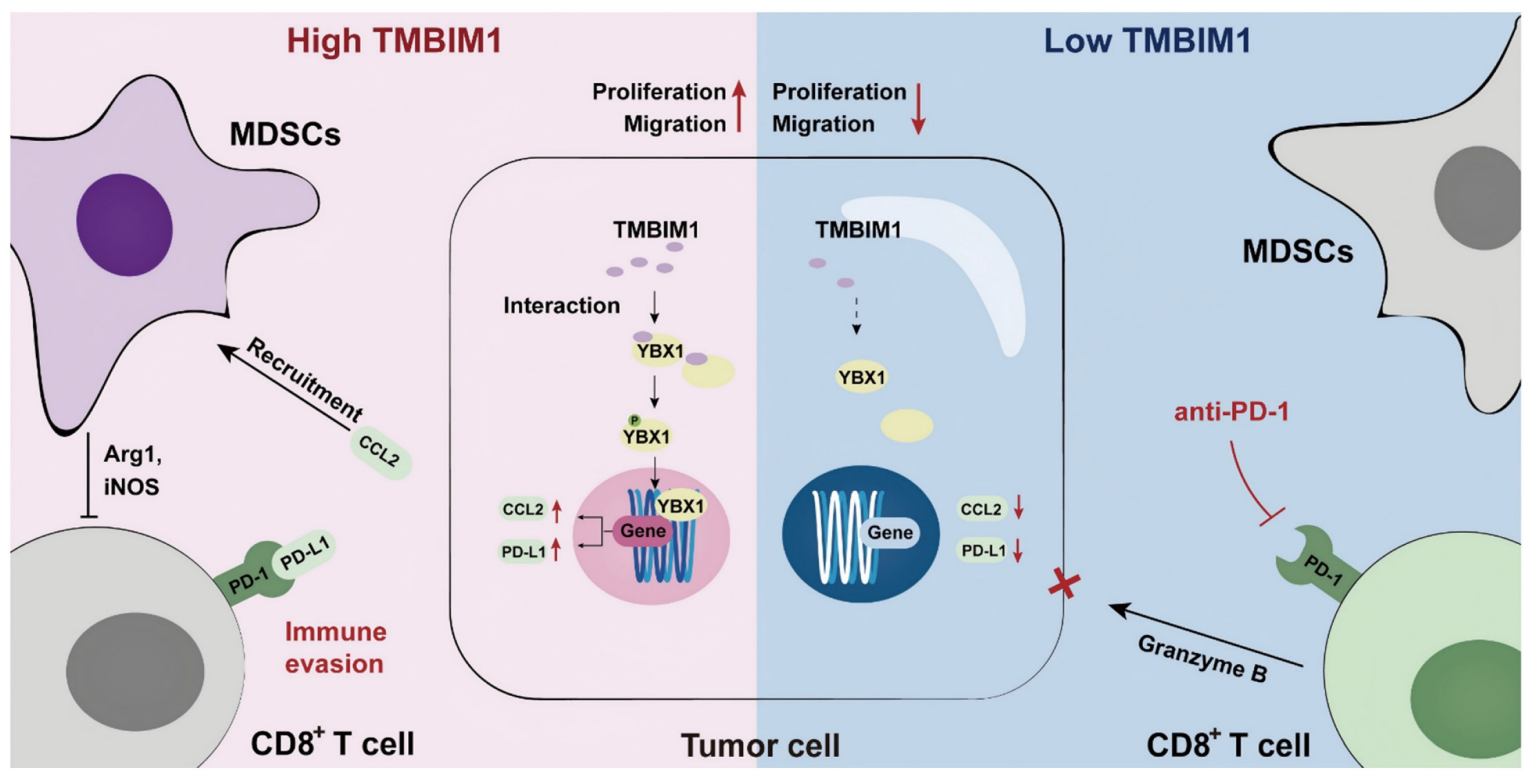
** Graphical abstract of TMBIM1's role in PDAC.** TMBIM1 promotes MDSC recruitment, immune evasion, and CCL2/PD-L1 expression via YBX1, while its suppression enhances CD8^+^ T cell activity and anti-PD-1 therapy efficacy.

On the basis of these findings, we propose a model in which TMBIM1 drives immune suppression within the pancreatic TME by increasing MDSC recruitment and upregulating the expression of immunosuppressive factors such as CCL2 and PD-L1. This leads to reduced infiltration and activation of CD8^+^ T cells, thus enabling immune escape and tumor progression. In contrast, knocking down TMBIM1 diminished these suppressive effects, reducing MDSC recruitment, increasing CD8^+^ T-cell activity, and increasing sensitivity to PD-1 blockade (Figure 8, right panel). Thus, targeting TMBIM1 could serve as a potential therapeutic approach to counteract pancreatic cancer resistance to ICBs.

## Discussion

PDAC is considered an immunologically 'cold' tumor characterized by poor infiltration of CD8^+^ T cells and an overall lack of response to ICB therapies, such as anti-PD-1 therapy 29. Additionally, the expression and distribution of PD-L1 in cells can minimize the therapeutic response to ICB-based treatments 30. Despite the low mutational burden and scarcity of targetable neoantigens in PDAC, emerging evidence suggests that immunotherapies can be effective when combined with approaches that modulate the TIME 31, 32. Our study highlights the importance of TMBIM1 in shaping the immune landscape of pancreatic cancer, demonstrating that its inhibition promotes CD8^+^ T-cell infiltration while reducing MDSC accumulation, thereby enhancing the effectiveness of PD-1 blockade.

A key finding of our study is the ability of TMBIM1 to promote MDSC recruitment in the PDAC microenvironment by inducing YBX1 transcription downstream of CCL2. MDSCs are a major component of the immunosuppressive milieu as they limit the activation and function of cytotoxic T cells 33. Previous reports have shown that CCL2 expression contributes to immune resistance by attracting MDSCs and tumor-associated macrophages 34, 35. By knocking down TMBIM1 in a pancreatic cancer model, we observed a significant reduction in MDSC infiltration, which was correlated with improved antitumor immune responses. The reduced recruitment of MDSCs in TMBIM1-knockdown tumors likely facilitates greater infiltration and activation of CD8^+^ T cells, particularly those expressing GZMB, a marker of cytotoxic activity 36, 37. Additionally, the significant upregulation of PD-L1 via the TMBIM1/YBX1 axis is indispensable for building an immunosuppressive TME in PDAC. These findings support the notion that targeting TMBIM1 can reprogram the TME to favor immune surveillance and tumor destruction. The impact of TMBIM1 inhibition on CD8^+^ T-cell activity is particularly noteworthy, as these cells are critical effectors of antitumor immunity. Our data demonstrate that TMBIM1 knockdown increases both the quantity and functionality of CD8^+^ T cells in tumors, as evidenced by their increased cytolytic activity. When combined with PD-1 blockade, TMBIM1 knockdown leads to even greater T-cell activation, suggesting a synergistic relationship between these two therapeutic strategies. These findings are consistent with other studies that suggest that effective CD8^+^ T-cell responses can be induced in PDAC through combination therapies, despite the inherent resistance of this tumor type to single-agent immunotherapies 38, 39.

The clinical relevance of TMBIM1 in pancreatic cancer is underscored by our survival analysis of both the TCGA and FUSCC cohorts, where high TMBIM1 expression was significantly associated with poor OS and PFS. These findings establish TMBIM1 as a robust negative prognostic marker in PDAC. Mechanistically, our data suggest that TMBIM1-mediated immune suppression is the dominant mechanism enabling PDAC immune evasion. This finding is consistent with growing evidence that immune evasion in PDAC is driven by an immunosuppressive microenvironment that inhibits effective CD8^+^ T-cell responses 40.

Our findings highlight the dual role of TMBIM1 in regulating immune cell recruitment and modulating the TIME. Specifically, TMBIM1 promotes the infiltration of immunosuppressive MDSCs while concurrently reducing the presence of CD8^+^ T cells in the TME. These changes collectively reinforce an immunosuppressive landscape that diminishes antitumor immune responses. Importantly, these observations suggest that targeting TMBIM1 could serve as a potential strategy to reprogram the TIME and restore effective immune surveillance.

Given the heterogeneity of PDAC and its complex immunosuppressive TME, it is essential to identify biomarkers that can predict responses to immune-based therapies 41. The interplay between TMBIM1 expression, MDSC recruitment, and T-cell activation provides a strong rationale for considering TMBIM1 as a therapeutic target in PDAC. Additionally, our findings suggest that TMBIM1 expression may serve as a predictive biomarker for patient selection in future clinical trials of combination treatments composed of immune checkpoint inhibitors and agents that target the TME.

## Conclusions

In conclusion, our study highlights the critical role of the TMBIM1/YBX1 axis in regulating the immunosuppressive TME in PDAC. We demonstrated that TMBIM1 promotes an immunosuppressive TME by driving MDSC recruitment, which suppresses antitumor immune responses and reduces the effectiveness of PD-1 checkpoint blockade. YBX1, a key transcriptional regulator that interacts with TMBIM1, was found to control the expression of CCL2 and PD-L1, further facilitating MDSC-mediated immune evasion. Clinically, elevated TMBIM1 expression is associated with poor patient outcomes and correlates with increased CCL2 and PD-L1 levels, underscoring its importance in modulating immune suppression in patients with PDAC. Our findings position the TMBIM1/YBX1 axis as a promising therapeutic target in PDAC, with the potential to reprogram the TIME and increase the efficacy of immunotherapy, providing a foundation for future research and clinical strategies.

## Supplementary Material

Supplementary figures and tables 1-5, 7.

Supplementary table 6.

## Figures and Tables

**Table 1:** Univariate and multivariate Cox regression analyses of overall survival in 169 PDAC patients with R0 margins at the FUSCC.

Variants	Hazard Ratio	95% CI	P-value	Hazard Ratio	95% CI	P-value
	Univariate Cox			Multivariate Cox		
**Age**						
>60 years	1.144	0.755 to 1.733	0.525			
≤60 years						
**Gender**						
Male	1.051	0.703 to 1.571	0.808			
Female						
**Tumor Location**						
Pancreatic Body-Tail	0.89	0.595 to 1.33	0.569			
Pancreatic Head						
**Nerve Invasion**						
Yes	1.563	0.682 to 3.581	0.291			
No						
**Vascular Cancer Emboli**						
Yes	1.61	1.076 to 2.409	**0.021**	1.379	0.9 to 2.112	0.14
No						
**LN Metastasis**						
Yes	1.525	1.024 to 2.271	**0.038**	1.237	0.808 to 1.893	0.328
No						
**Preoperative CA19-9 Value**						
<=230 U/ml	1.771	1.187 to 2.642	**0.005**	1.424	0.937 to 2.163	0.098
>230 U/ml						
**Tumor Size**						
≥3 cm	1.911	1.276 to 2.861	**0.002**	1.311	0.85 to 2.022	0.22
<3 cm						
**T Stage**						
II-III	4.146	1.522 to 11.297	**0.005**	3.311	1.163 to 9.427	**0.025**
I						
**IHC Score**						
High (≥ 6 points)	1.846	1.235 to 2.759	**0.003**	1.844	1.224 to 2.778	**0.003**
Low (< 6 points)						

## Abbreviations

PDAC: Pancreatic ductal adenocarcinoma
TIME: Tumor immune microenvironment
TMBIM1: Transmembrane BAX Inhibitor Motif Containing 1
MDSC: Myeloid-derived suppressor cell
YBX1: Y box binding protein 1
OS: Overall survival
ICB: Immune checkpoint blockade
CTLs: Cytotoxic T lymphocytes
PMN-MDSCs: Polymorphonuclear MDSCs
M-MDSCs: Monocytic MDSCs
Arg1: Arginase 1
CCL2: Chemokine ligand 2
TMA: Tissue microarray
FUSCC: Fudan University Shanghai Cancer Center
HPDE: Human pancreatic ductal cells
GEO: Gene Expression Omnibus
qPCR: Real-time quantitative PCR
PFA: Paraformaldehyde
Co-IP: Coimmuno-precipitation
ChIP: Chromatin immuno-precipitation
IHC: Immunohistochemistry
SD: Standard deviation
HR: Hazard ratio
ROC: Receiver operating characteristic
TCGA-PAAD: The Cancer Genome Atlas-Pancreatic Adenocarcinoma
FUSCC: Fudan University Shanghai Cancer Center
RNA-seq: RNA-sequencing
scRNA-seq: Single cell RNA-seq
CCL2-OE: Overexpression of CCL2
rCCL2: Recombinant CCL2
αCCL2: Anti-CCL2 antibody
IP: Immunoprecipitation
LC-MS: Liquid chromatography-mass spectrometry
WT: Wild-type
MUT: Mutant
PFS: Progression-free survival
