# Supplementary material for: The TMBIM1-YBX1 axis orchestrates MDSC recruitment and immunosuppressive microenvironment in pancreatic cancer
Source: Theranostics. 2025 Feb 3;15(7):2794–813. doi: 10.7150/thno.111180 (PMC11898282; doi:10.7150/thno.111180)
Supplement: Supplementary file 1 — Supplementary figures and tables 1-5, 7. [file thnov15p2794s1.pdf]

Table S1. Sequences of knocked-down genes.

Name	Sequence (5'-3')
shTMBIM1#1	CCGTTTCCCATGGAACATCAT
shTMBIM1#2	GCTGTCTACTACGTGTCCTAT
siYBX1#1	GGAGGCAGCAAAUGUUACA
siYBX1#2	UGUAACAUUUGCUGCCUCC
siYBX1#3	CCACGCAAUUACCAGCAAA

Table S2. Coding Sequences for Overexpression Plasmids (TMBIM1 and CCL2)

Name	Sequence (5'-3')
TMBIM1-Flag	gaattcATGTCCAACCCCAGCGCCCCACCACCATATGAAGACCGCAAC<br>CCCCTGTACCCAGGCCCTCCGCCCCCTGGGGGCTATGGGCAGCCAT<br>CTGTCCTGCCAGGAGGGTATCCTGCCTACCCTGGCTACCCGCAGCC<br>TGGCTACGGTCACCCTGCTGGCTACCCACAGCCCATGCCCCCCACC<br>CACCCGATGCCCATGAACTACGGCCCAGGCCATGGCTATGATGGG<br>GAGGAGAGAGCAGTGAGTGATAGCTTCGGGCCTGGAGAGTGGGAT<br>GACCGGAAAGTGCGACACACTTTTATCCGAAAGGTTTACTCCATCA<br>TCTCCGTGCAGCTGCTCATCACTGTGGCCATCATTGCTATCTTCACC<br>TTTGTGGAACCTGTCAGCGCCTTTGTGAGGAGAAATGTGGCTGTCT<br>ACTACGTGTCCTATGCTGTCTTCGTTGTCACCTACCTGATCCTTGCC<br>TGCTGCCAGGGACCCAGACGCCGTTTCCCATGGAACATCATTCTGC<br>TGACCCTTTTTACTTTTGCCATGGGCTTCATGACGGGCACCATTTCC<br>AGTATGTACCAAACCAAAGCCGTCATCATTGCAATGATCATCACTG<br>CGGTGGTATCCATTTCAGTCACCATCTTCTGCTTTCAGACCAAGGT<br>GGACTTCACCTCGTGCACAGGCCTCTTCTGTGTCCTGGGAATTGTG<br>CTCCTGGTGACTGGGATTGTCACTAGCATTGTGCTCTACTTCCAAT<br>ACGTTTACTGGCTCCACATGCTCTATGCTGCTCTGGGGGCCATTTGT<br>TTCACCCTGTTCCTGGCTTACGACACACAGCTGGTCCTGGGGAACC<br>GGAAGCACACCATCAGCCCCGAGGACTACATCACTGGCGCCCTGC<br>AGATTTACACAGACATCATCTACATCTTCACCTTTGTGCTGCAGCT<br>GATGGGGGATCGCAATGATTACAAGGATGACGACGATAAGTAAgga<br>tcc
CCL2	gaattcATGAAAGTCTCTGCCGCCCTTCTGTGCCTGCTGCTCATAGCAG<br>CCACCTTCATTCCCCAAGGGCTCGCTCAGCCAGATGCAATCAATGCC<br>CCAGTCACCTGCTGTTATAACTTCACCAATAGGAAGATCTCAGTGCA<br>GAGGCTCGCGAGCTATAGAAGAATCACCAGCAGCAAGTGTCCCAA<br>AGAAGCTGTGATCTTCAAGACCATTGTGGCCAAGGAGATCTGTGCT<br>GACCCCAAGCAGAAGTGGGTTCAGGATTCCATGGACCACCTGGAC<br>AAGCAAACCCAAACTCCGAAGACTTGAggatcc

Table S3. Primers used

Name	Sequence (5'- 3')
β-ACTIN-F	CATGTACGTTGCTATCCAGGC
β-ACTIN-R	CTCCTTAATGTCACGCACGAT
TMBIM1-F	GAGAGAGCGGTGAGTGATAGC
TMBIM1-R	ACCTTTCGGATAAAAGTGTGTCG
CD274-F	GCTGCACTAATTGTCTATTGGGA
CD274-R	AATTCGCTTGTAGTCGGCACC
CCL2-F	CAGCCAGATGCAATCAATGCC
CCL2-R	TGGAATCCTGAACCCACTTCT
PD-L1-Chip-M1F1	CCAACTTCGGGAACTTTGGG
PD-L1-Chip-M1R1	GCATGGAGTTCTCTTTGGCC
PD-L1-Chip-M1F2	CACCCAAACTTACAGTCACCA
PD-L1-Chip-M1R2	GCTGACACTGCCTTGATTTG
PD-L1-Chip-M1F3	CTTACAGTCACCAAAATTGCTCT
PD-L1-Chip-M1R3	TGGGAAATTATTGAGGCTGACAC
PD-L1-Chip-M2F1	TTTGGAAGCAAATGTCATAACCA
PD-L1-Chip-M2R1	CCTGTGTGCTCCCTTTTCTT
PD-L1-Chip-M2F2	TGGAAGCAAATGTCATAACCAAT
PD-L1-Chip-M2R2	CCTCAAGTGATCCGCCAAAG
PD-L1-Chip-M2F3	GTGTCAGCCTCAATAATTTCCCA
PD-L1-Chip-M2R3	TGGACTCTTCGTTGTTTGCC
PD-L1-Chip-M1F1	AGGAAAGGCAAACAACGAAGA
PD-L1-Chip-M1R1	GGTGCTCTCTTTTCTCGAACTC
PD-L1-Chip-M1F2	GGCAAACAACGAAGAGTCCA
PD-L1-Chip-M1R2	TATCCCGCGCTGAACTTCTA
PD-L1-Chip-M1F3	AAGAAAAGGGAGCACACAGG
PD-L1-Chip-M1R3	AATGGGCCCAAGATGACAGA
CCL2-Chip-M1F1	ACACTGGTTGGGGAGAAAAG
CCL2-Chip-M1R1	AGTGGCCATTTCAGACTCTG
CCL2-Chip-M1F2	TGGTTGGGGAGAAAAGGAGT
CCL2-Chip-M1R2	GAGAATCCCAGAGCAGAGACT
CCL2-Chip-M1F3	GGAGTAACTAGTGAGATTCAGGC
CCL2-Chip-M1R3	GAGAATCCCAGAGCAGAGACT
CCL2-Chip-M1F4	GCAGGGCTCGAGTTGATTTG
CCL2-Chip-M1R4	TCTCTTCTTCCTGGGACTAGAC
CCL2-Chip-M2F1	AGTTTCCTCGCTTCCTTCCT
CCL2-Chip-M2R1	TCCATTCACTGCTGAGACCA
CCL2-Chip-M2F2	CTGCAGTTTTCGCTTCAGAGA
CCL2-Chip-M2R2	AGTAGGAAAGGGAAGCAGGG
CCL2-Chip-M2F3	TGTGGTCAGTCTGGGCTTAA
CCL2-Chip-M2R3	TCCATGAGTGATAAGTGGGCT
CCL2-Chip-M2F4	TGTGGTCAGTCTGGGCTTAA
CCL2-Chip-M2R4	CAAGCAGGAGGAGGGATCTT
CCL2-Chip-M3F1	TAATGCATTGTCAGGGAGCC

CCL2-Chip-M3R1	TGCAGAAAAGGAAGGAAGCG
CCL2-Chip-M3F2	TAATGCATTGTCAGGGAGCC
CCL2-Chip-M3R2	TTTCTCTGAAGCGAAAACTGC
CCL2-Chip-M3F3	CCTTGGAATGTGGCCTGAAG
CCL2-Chip-M3R3	TTAAGCCCAGACTGACCACA
musACTB-F	GGCTGTATTCCCCTCCATCG
musACTB-R	CCAGTTGGTAACAATGCCATGT
musINOS-F	GTTCTCAGCCCAACAATACAAGA
musINOS-R	GTGGACGGGTCGATGTCAC
musARG1-F	CTCCAAGCCAAAGTCCTTAGAG
musARG1-R	AGGAGCTGTCATTAGGGACATC
musCCL2-F	TTAAAAACCTGGATCGGAACCAA
musCCL2-R	GCATTAGCTTCAGATTTACGGGT
musTMBIM1-F	AATCTTCACCTTTGTGGAACCAG
musTMBIM1-R	CAGGCAAGGGTCAGGTAGG
musCD274-F	GCTCCAAAGGACTTGTACGTG
musCD274-R	TGATCTGAAGGGCAGCATTTC

Table S4. Antibodies used.

Name	Source	Identifier
TMBIM1	Abcam	ab121358
CCL2	Abclonal	A23288
CCL2	Proteintech	66272-1-Ig
PD-L1	Abcam	ab205921
β-ACTIN	Proteintech	HRP-66009
a-TUBULIN	Proteintech	11224-1-AP
Flag	Sigma	F1802
P-YBX1	CST	2900
YBX1	Santacruz	sc-101198
Lamin b1	Proteintech	12987-1-AP
CD33	Abcam	ab270942
CD11B	Proteintech	66519-1-Ig
Ki67	Abcam	ab15580
anti-mouse PD-1	BioXCell	BE0146
IgG2a	BioXCell	BE0146
InVivoMAb anti-mouse/human/rat CCL2 (MCP-1)	BioXCell	BE0185
APC-Cy7 Rat Anti-Mouse CD45(30-F11)	BD Pharmingen	557659
Alexa Fluor 700 Hamster anti-Mouse CD3e(500A2)	BD Pharmingen	557984
FITC Rat Anti-Mouse CD8a (53-6.7)	BD Pharmingen	553030
PE anti-human/mouse Granzyme B Recombinant	Biolegend	396406
Purified Rat Anti-Mouse CD16/CD32 (Mouse BD Fc Block) (2.4G2)	BD Pharmingen	553141
PerCP-Cy5.5 Rat Anti-CD11b(M1/70)	BD Pharmingen	550993
PE-Cy7 Rat Anti-Mouse CD4(RM4-5)	BD Pharmingen	552775
PE CD33 WM53	BD Pharmingen	555450
Ms Ly-6G Ly-6C BV650 RB6-8C5 50ug	BD Pharmingen	740454

Table S5. ELISA kits used.

Name	Source	Identifier
CCL2 ELISA human	JINGMEI BIOTECHNOLOGY	JM-1401H1
CCL2 ELISA mouse	JINGMEI BIOTECHNOLOGY	JM-03003M2
PD-L1 ELISA human	JINGMEI BIOTECHNOLOGY	JM-5676H1
PD-L1 ELISA mouse	JINGMEI BIOTECHNOLOGY	JM-11722

Table S7. Molecular docking results.

TMBIM1(red)	YBX1(green)
ASN-3	THR-7
GLN-44	ARG-282
GLY-46	ARG-282
ALA-51	ARG-279
GLN-55	ARG-247
PRO-56	ARG-247
PRO-54	ARG-253
PRO-62	TYR-241
MET-65	TYR-238

Figure S1

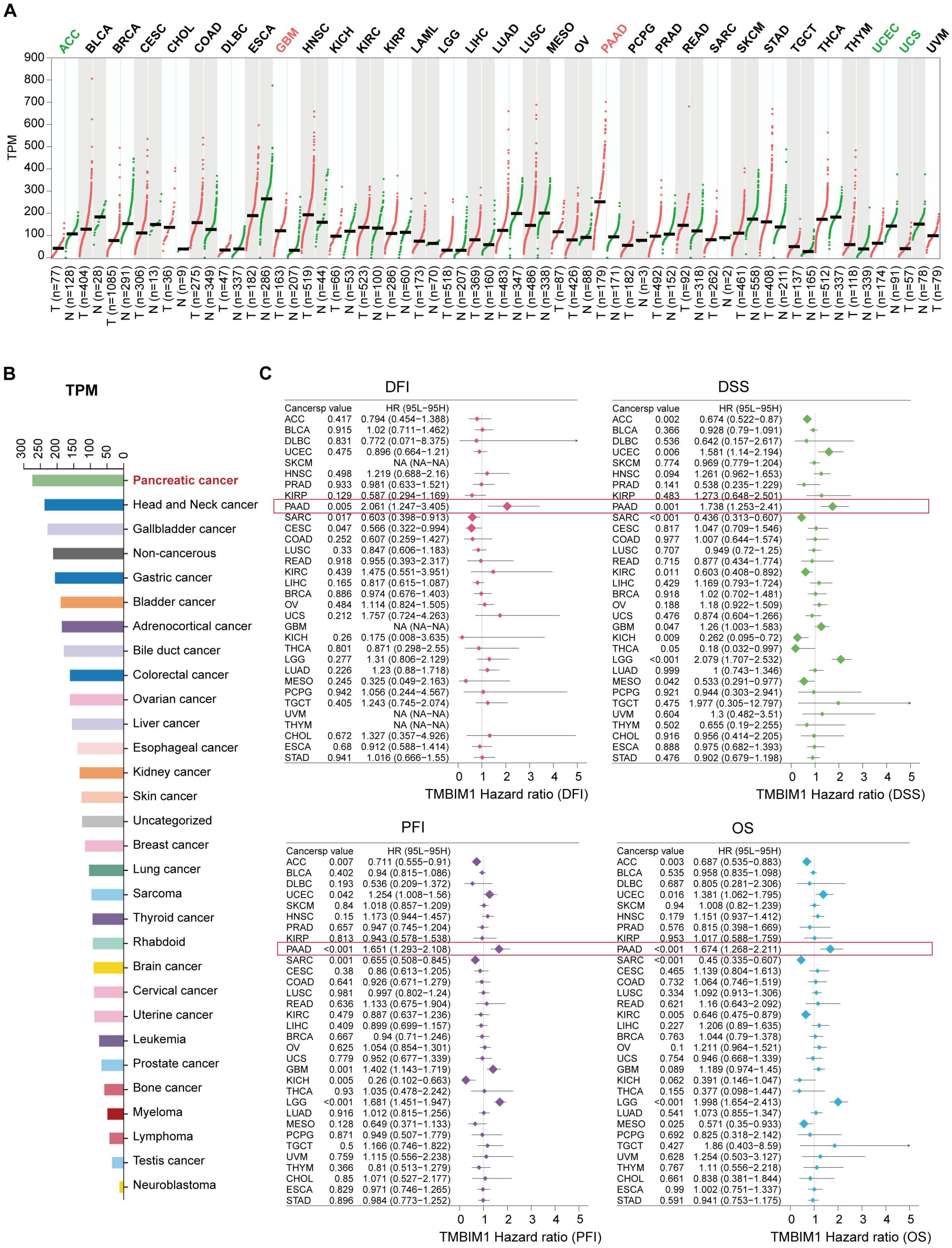

TMBIM1 expression levels and prognostic significance across various cancer types.

(A) TMBIM1 expression across different cancer types based on TCGA dataset. The box plots represent the transcriptional expression of TMBIM1 (TPM) in tumor (T) and adjacent normal (N) tissues. Tumor types

with significant differences in TMBIM1 expression are highlighted in green (decreased in tumor) and red (increased in tumor) compared to normal tissues ($P < 0.05$). (B) TMBIM1 expression across pancreatic cancer and other selected cancer cell lines from the HPA database. Pancreatic cancer cells exhibit some of the highest TMBIM1 expression levels compared to other cancer types, as indicated by TPM values. (C) Forest plots showing the prognostic value of TMBIM1 expression in various cancers in TCGA database for Disease-Free Interval (DFI), Disease-Specific Survival (DSS), Progression-Free Interval (PFI), and Overall Survival (OS). Hazard ratios (HR) with 95% confidence intervals (CI) were calculated for each cancer type. Higher TMBIM1 expression is significantly associated with worse prognosis in multiple cancers, including pancreatic cancer, as indicated by the red boxes. Statistical significance is marked by P-values < 0.05.

Figure S2

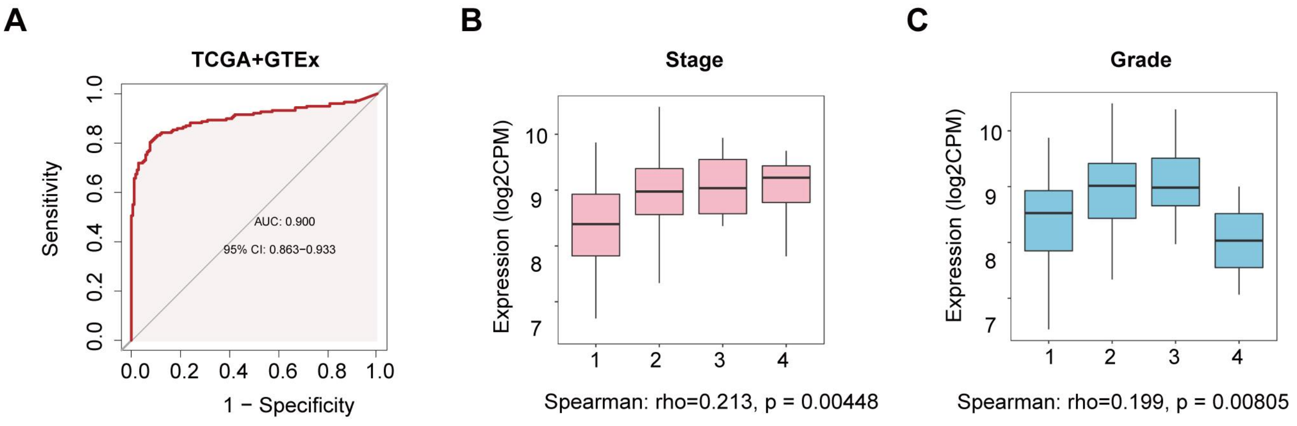

TMBIM1 expression and diagnostic significance in pancreatic cancer.

(A) ROC curve analysis of TMBIM1 expression for distinguishing pancreatic cancer from normal tissues using TCGA and GTEx data (AUC = 0.900, 95% CI: 0.863–0.933). (B) Boxplot showing TMBIM1 expression across different tumor stages in TCGA-PAAD, indicating a positive correlation (Spearman: rho = 0.213, p = 0.00448). (C) Boxplot showing TMBIM1 expression across different tumor grades in TCGA-PAAD (Spearman: rho = 0.199, p = 0.00805).

Figure S3

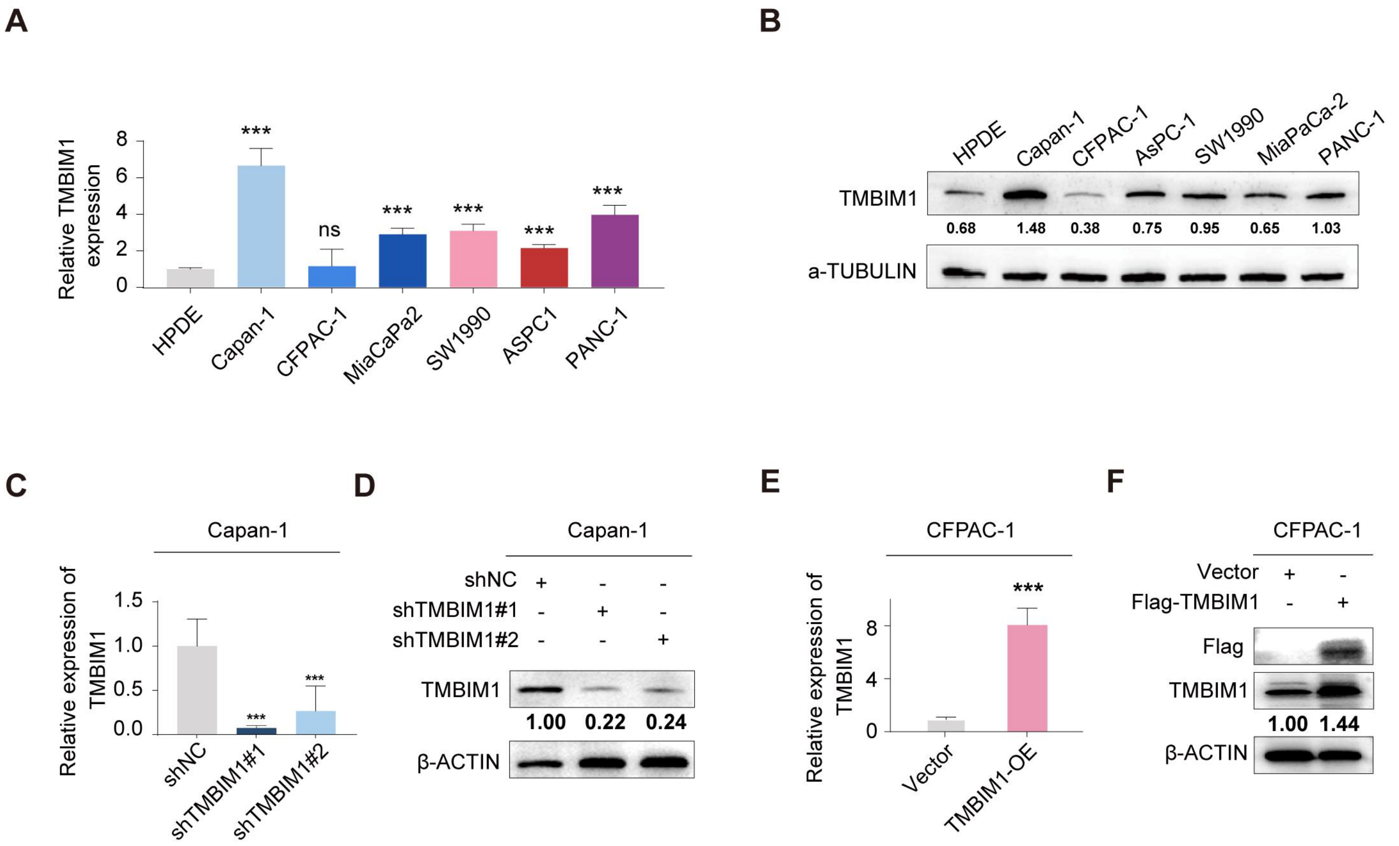

Quantification of mRNA and protein expression of TMBIM1 in pancreatic cancer cell lines and stably transfected cell lines.

(A-B) Relative TMBIM1 mRNA (A) and protein (B) expression levels in human pancreatic cell lines (HPDE, Capan-1, CFPAC-1, MiaPaCa-2, SW1990, AsPC-1, and PANC-1) compared with HPDE cells as a control. tatistical significance was determined using Student's t test. (F). (C-D) Validation of TMBIM1 knockdown efficiency in Capan-1 shRNA constructs (shTMBIM1#1 and shTMBIM1#2) at the mRNA levels (C) and protein (D). (E-F) Overexpression of TMBIM1 in CFPAC-1 cells transfected with a Flag-TMBIM1 vector, confirmed at the mRNA levels (E) and protein (F). Statistical significance was determined using Student's t test (C, E). The data are presented as the mean ± standard deviation (SD). *P < 0.05; **P < 0.01; ***P < 0.001; ns, not significant.

Figure S4

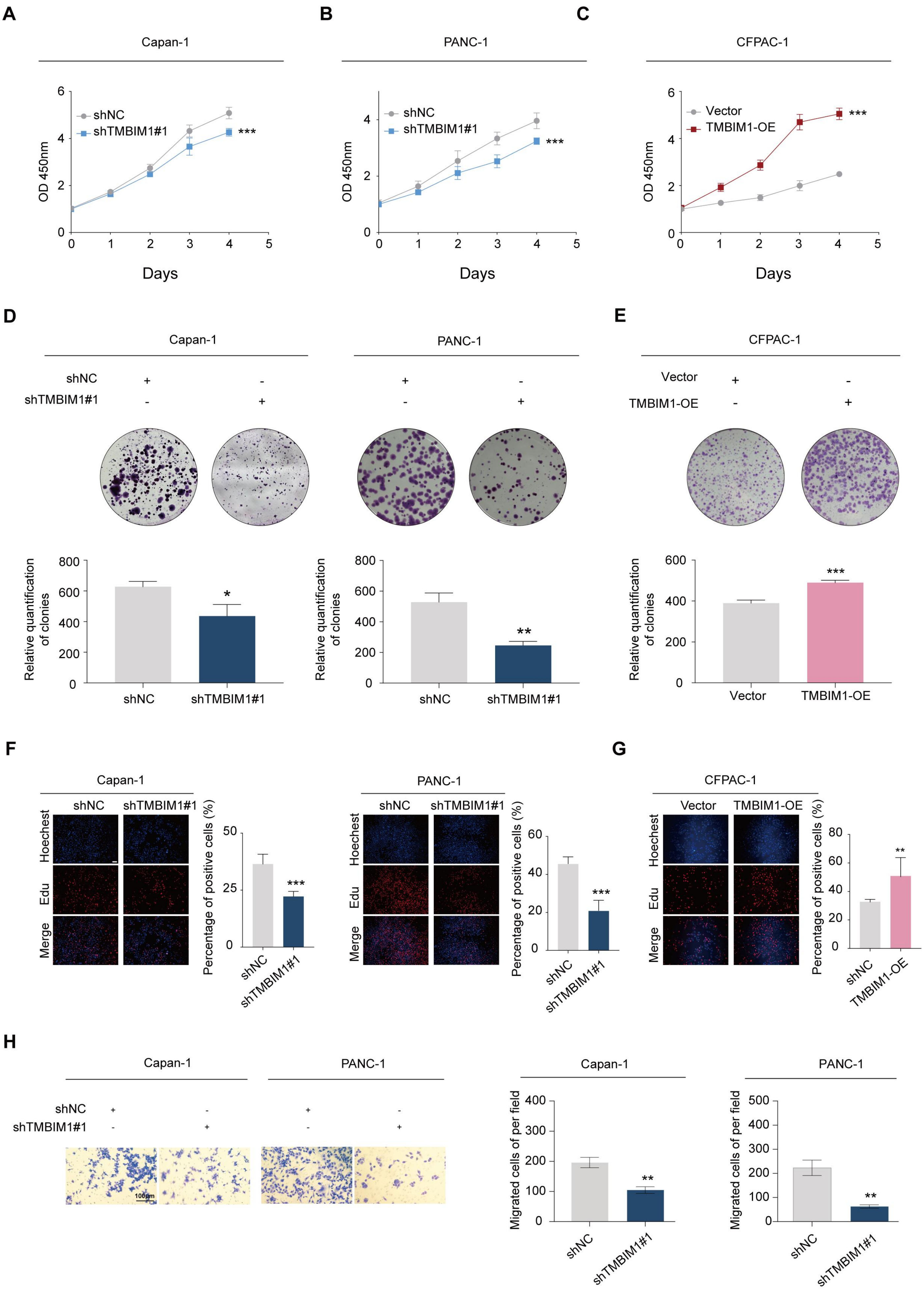

A
Capan-1
OD 450nm
shNC
shTMBIM1#1
***
Days
B
PANC-1
C
CFPAC-1
Vector
TMBIM1-OE
D
E
Relative quantification of clonies
*
**
F
G
Hoechest
Edu
Merge
Percentage of positive cells (%)
H
Migrated cells of per field
100μm

53

Functional assays assessing the impact of TMBIM1 in pancreatic cancer cell lines.

(A-C) CCK8 assays measuring cell viability of Capan-1, PANC-1, and CFPAC1 cells following treatment with TMBIM1 knockdown or overexpression were performed over 4 days. (D) Colony formation assays in Capan-1 and PANC-1 cells, comparing shNC and shTMBIM1. (E) Colony formation assays in CFPAC1 cells, comparing vector control and TMBIM1-OE. (F-G) Edu incorporation assays evaluating DNA synthesis and cell proliferation in Capan-1, PANC-1, and CFPAC1 cells, scale bar, 100 μm. (H) Transwell migration assays measuring the migratory ability of Capan-1, PANC-1 following treatment with shTMBIM1, scale bar, 100 μm. All assays were performed in triplicates, with statistical significance determined by Student's t-test and indicated for each comparison to the vector control group. The data are presented as the means ± SDs.(n.s., not significant, *$p < 0.05$, **$p < 0.01$, ***$p < 0.001$).

Figure S5.

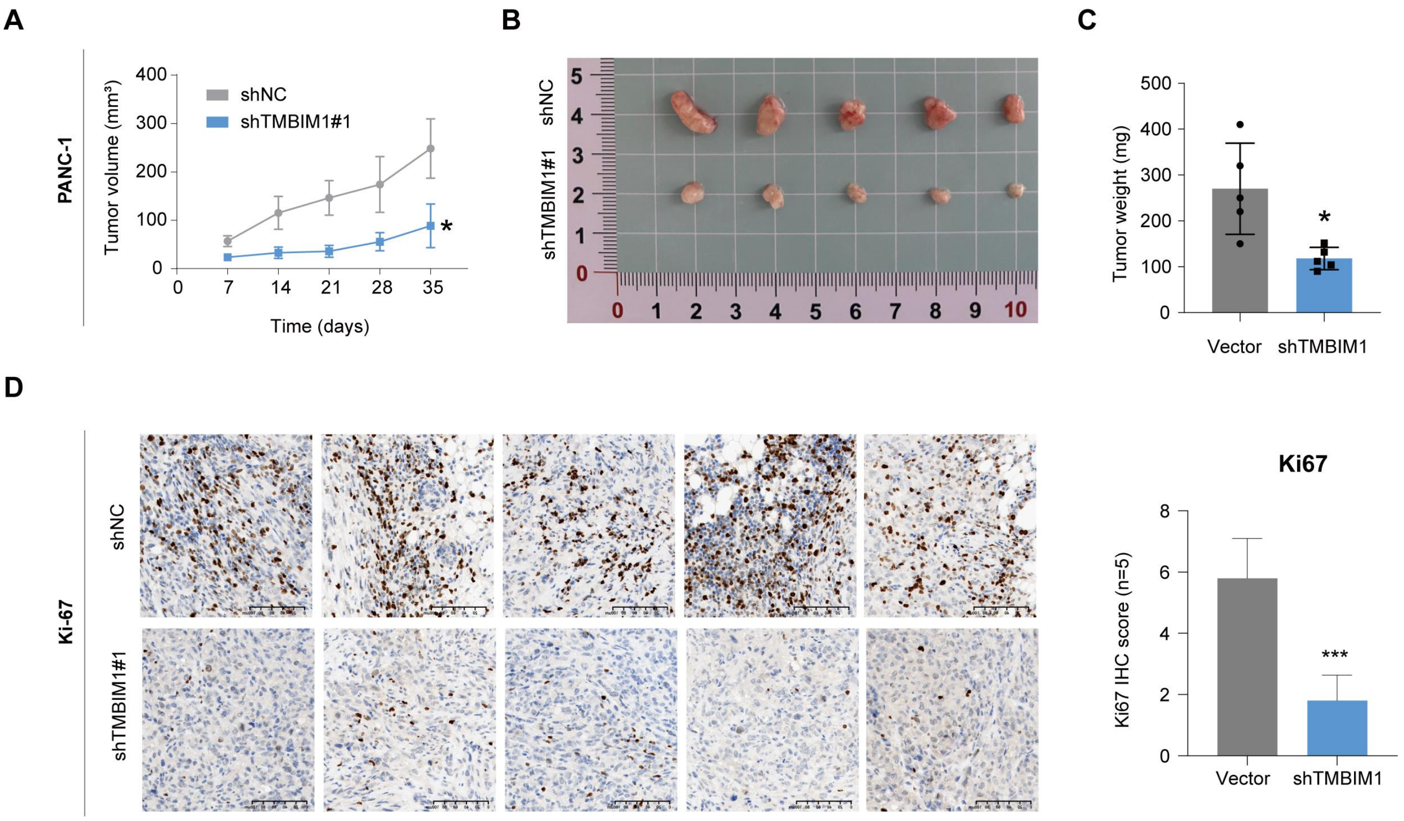

Knockdown of TMBIM1 inhibits pancreatic tumor growth in vivo.

(A) Tumor volume in mice injected with PANC-1 cells transfected with either shNC (control) or shTMBIM1 was measured over 35 days. (B) Representative images of excised tumors from the shNC and shTMBIM1 groups.(C) Tumor weight comparison between control (vector) and shTMBIM1 groups. (D) Representative Ki67 IHC staining of tumor sections, showing reduced proliferation in shTMBIM1 tumors compared to controls, scale bar, 100 μm. Quantification of Ki67 IHC scores is shown on the right. Data are presented as mean ± SD. *p<0.05, ***p<0.001.

Figure S6

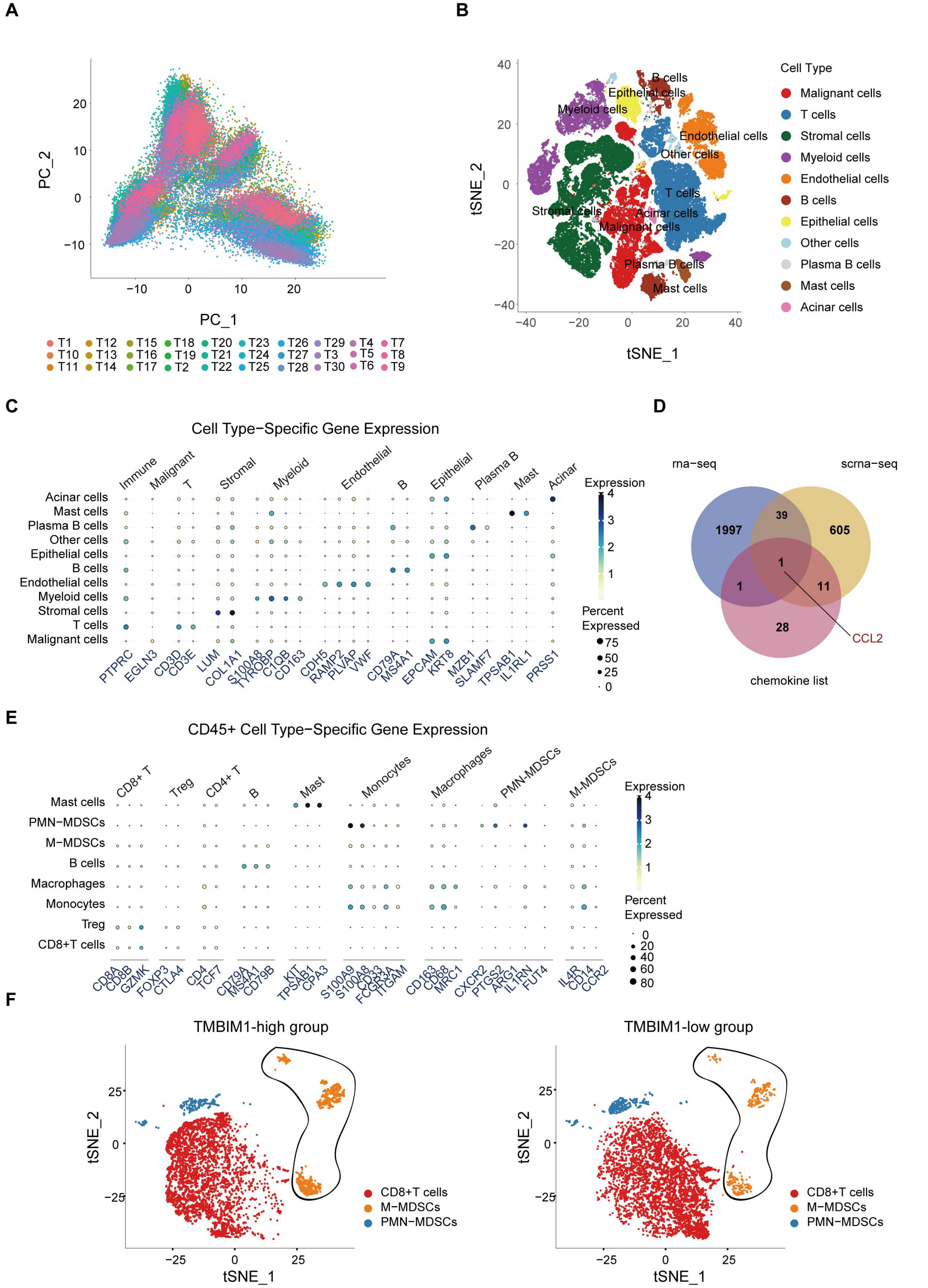
A
B
PC_2
PC_1
T1 T12 T15 T18 T20 T23 T26 T29 T4 T7
T10 T13 T16 T19 T21 T24 T27 T3 T5 T8
T11 T14 T17 T2 T22 T25 T28 T30 T6 T9
tSNE_2
tSNE_1
Cell Type
Malignant cells
T cells
Stromal cells
Myeloid cells
Endothelial cells
B cells
Epithelial cells
Other cells
Plasma B cells
Mast cells
Acinar cells
C
Cell Type−Specific Gene Expression
Immune Malignant T Stromal Myeloid Endothelial B Epithelial Plasma B Mast Acinar
Acinar cells
Mast cells
Plasma B cells
Other cells
Epithelial cells
B cells
Endothelial cells
Myeloid cells
Stromal cells
T cells
Malignant cells
PTPRC EGLN3 CD3D CD3E LUM COL1A1 S100A8 TYROBP C1QB CD163 CDH5 RAMP2 PLVAP VWF CD79A MS4A1 EPCAM KRT8 MZB1 SLAMF7 TPSAB1 IL1RL1 PRSS1
Expression
Percent Expressed
D
rna−seq
scrna−seq
1997
39
605
1
1
11
28
CCL2
chemokine list
E
CD45+ Cell Type−Specific Gene Expression
CD8+ T Treg CD4+ T B Mast Monocytes Macrophages PMN-MDSCs M-MDSCs
Mast cells
PMN−MDSCs
M−MDSCs
B cells
Macrophages
Monocytes
Treg
CD8+T cells
CD8A CD8B GZMK FOXP3 CTLA4 CD4 TCF7 CD79A MS4A1 CD79B KIT TPSAB1 CPA3 S100A9 S100A8 CD33 FCGR3A ITGAM CD163 CD68 MRC1 CXCR2 PTGS2 ARG1 IL1RN FUT4 IL4R CD14 CCR2
Expression
Percent Expressed
F
TMBIM1-high group
TMBIM1-low group
tSNE_2
tSNE_1
CD8+T cells
M−MDSCs
PMN−MDSCs

75

Multi-Dimensional analysis of cell types, gene expression, and TMBIM1-driven immune microenvironment in pancreatic cancer

Principal component analysis plot displaying the overall transcriptomic differences between cell types in the PDAC tumor microenvironment, based on CRA001160 and GSE212966. (B) t-SNE plot of PDAC tissue displaying clustering of distinct cell types, including T cells, MDSCs, macrophages, and malignant cells, on the basis of their transcriptomic profiles. (C) Dot plot showing cell type-specific gene expression across various cell populations, including immune, stromal, and malignant cell types, in PDAC, highlighting the distribution of key markers. (D) A Venn diagram illustrating the overlap of genes identified from bulk RNA-seq, single-cell RNA-seq (scRNA-seq), and a curated chemokine list (from TISDB). A total of 40 genes were shared between RNA-seq and scRNA-seq, while only one gene, CCL2, was found to be common across all three datasets, highlighting its potential significance in the tumor microenvironment of pancreatic cancer. (E) Dot plot showing cell type-specific gene expression across various cell populations, including mast, PMN-MDSCs, M-MDSCs, B, Macrophages, Monocytes, Treg, and $CD8^+$ T cells in all $CD45^+$ cells. (F) tSNE analysis illustrating immune cell distribution in TMBIM1-high and TMBIM1-low groups in pancreatic cancer ($CD8^+$ T cells (red), M-MDSCs (orange), and PMN-MDSCs (blue) in the TMBIM1-high group (left panel) and TMBIM1-low group (right panel)).

Figure S7

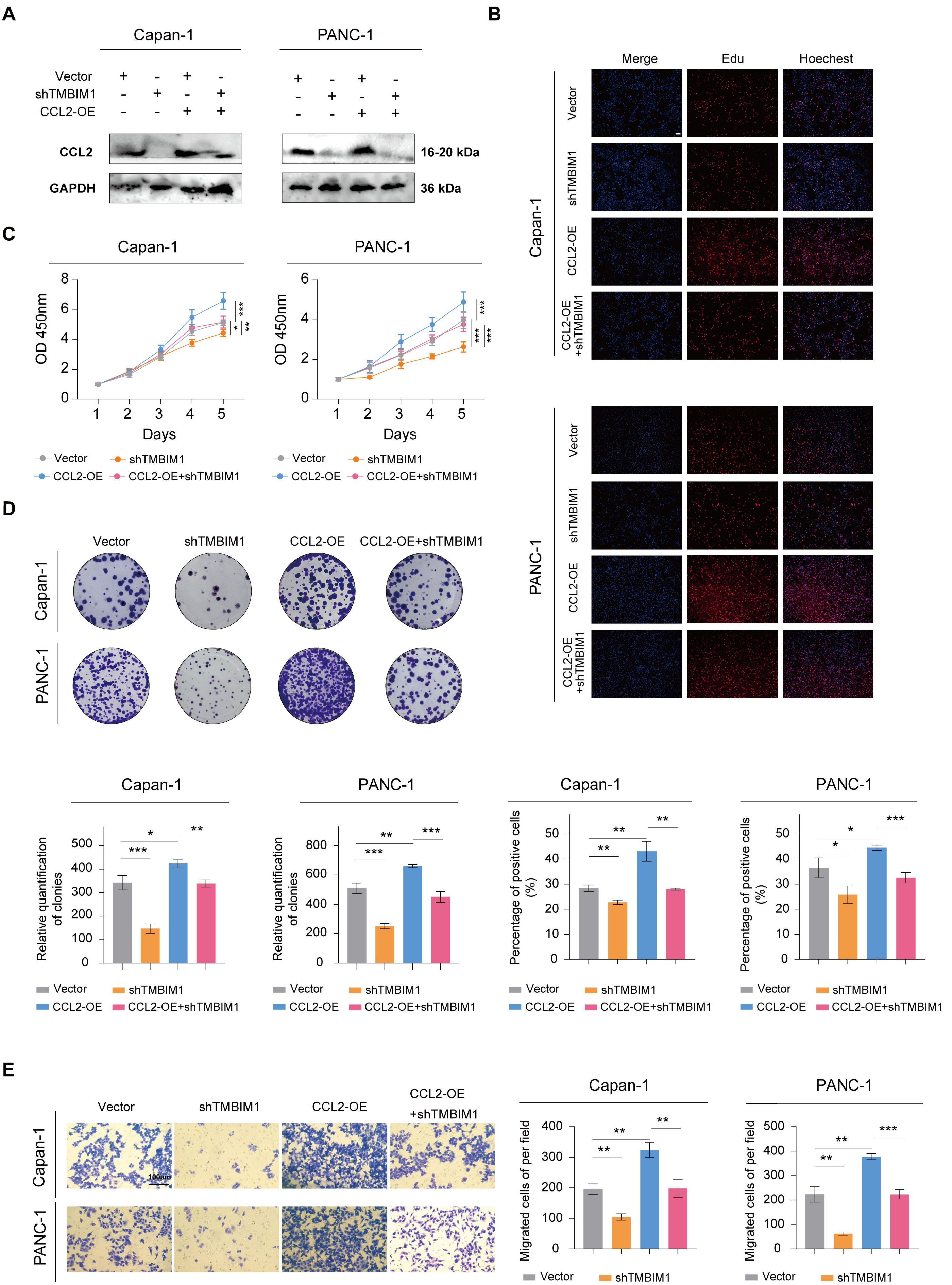
A
Capan-1
PANC-1
Vector
shTMBIM1
CCL2-OE
CCL2
GAPDH
16-20 kDa
36 kDa
B
Merge
Edu
Hoechest
Capan-1
PANC-1
Vector
shTMBIM1
CCL2-OE
CCL2-OE +shTMBIM1
C
OD 450nm
Days
Vector
shTMBIM1
CCL2-OE
CCL2-OE+shTMBIM1
D
Relative quantification of clonies
Percentage of positive cells (%)
E
Migrated cells of per field
100μm

94

Functional analysis of CCL2 overexpression (CCL2-OE) and shTMBIM1 in pancreatic cancer cells. (A) Western blot analysis of CCL2 and GAPDH expression in Capan-1 and PANC-1 cells treated with vector control (SHT), TMBIM1 knockdown (BLAM1), CCL2 overexpression (CCL2-OE), and combined TMBIM1 knockdown and CCL2 overexpression (BLAM1 + CCL2-OE). (B) EDU incorporation assays to assess cell proliferation in Capan-1 and PANC-1 cells across the four treatment groups, scale bar, 100 μm. (C) CCK8 assays evaluating cell viability in the four treatment groups for both Capan-1 and PANC-1 cells. (D) Colony formation assays assessing proliferative capacity of Capan-1 and PANC-1 cells in the four treatment groups. (E) Transwell migration assays measuring the migratory ability of Capan-1 and PANC-1 cells in the four treatment groups, scale bar, 100 μm. All assays were performed in triplicates, with statistical significance determined by Student's t-test and indicated for each comparison to the vector control group. The data are presented as the means ± SDs.(n.s., not significant, $*p < 0.05$, $**p < 0.01$, $***p < 0.001$).

Figure S8

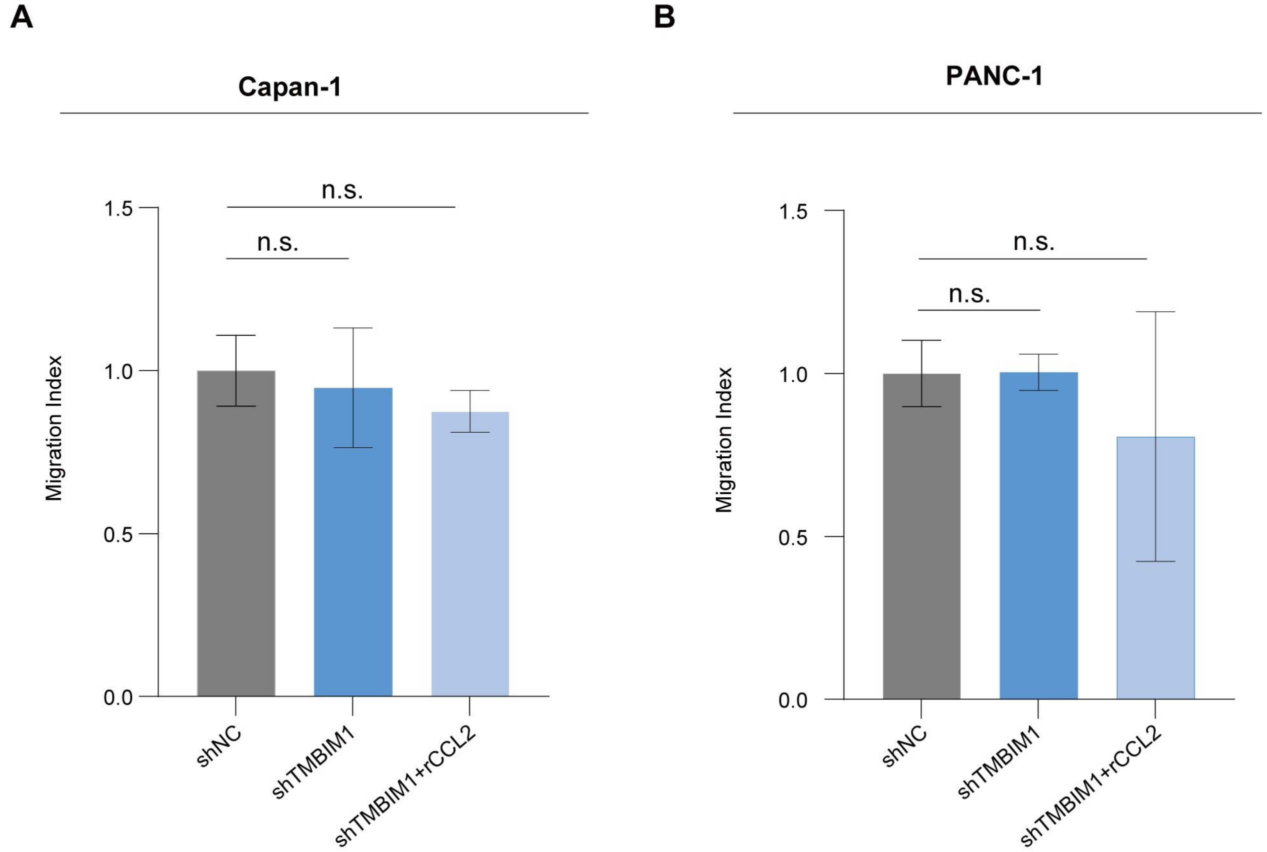

CD8$^+$ T cell migration in co-culture with tumor cells: Impact of TMBIM1 knockdown and rCCL2 supplementation.

(A-B) Migration of CD8$^+$ T cells was assessed in Capan-1 tumor cell groups (A) and PANC1 tumor cell group (B) under three conditions: control (NC), shTMBIM1, and shTMBIM1 + recombinant CCL2 (rCCL2). Migration was assessed using a transwell assay. Data are presented as mean±SD. No significant differences in T-cell migration were observed among the three groups, despite triplicate experiments. Statistical analysis was performed using a Student's t-test. (n.s., not significant, *$p < 0.05$, **$p < 0.01$, ***$p < 0.001$).

Figure S9

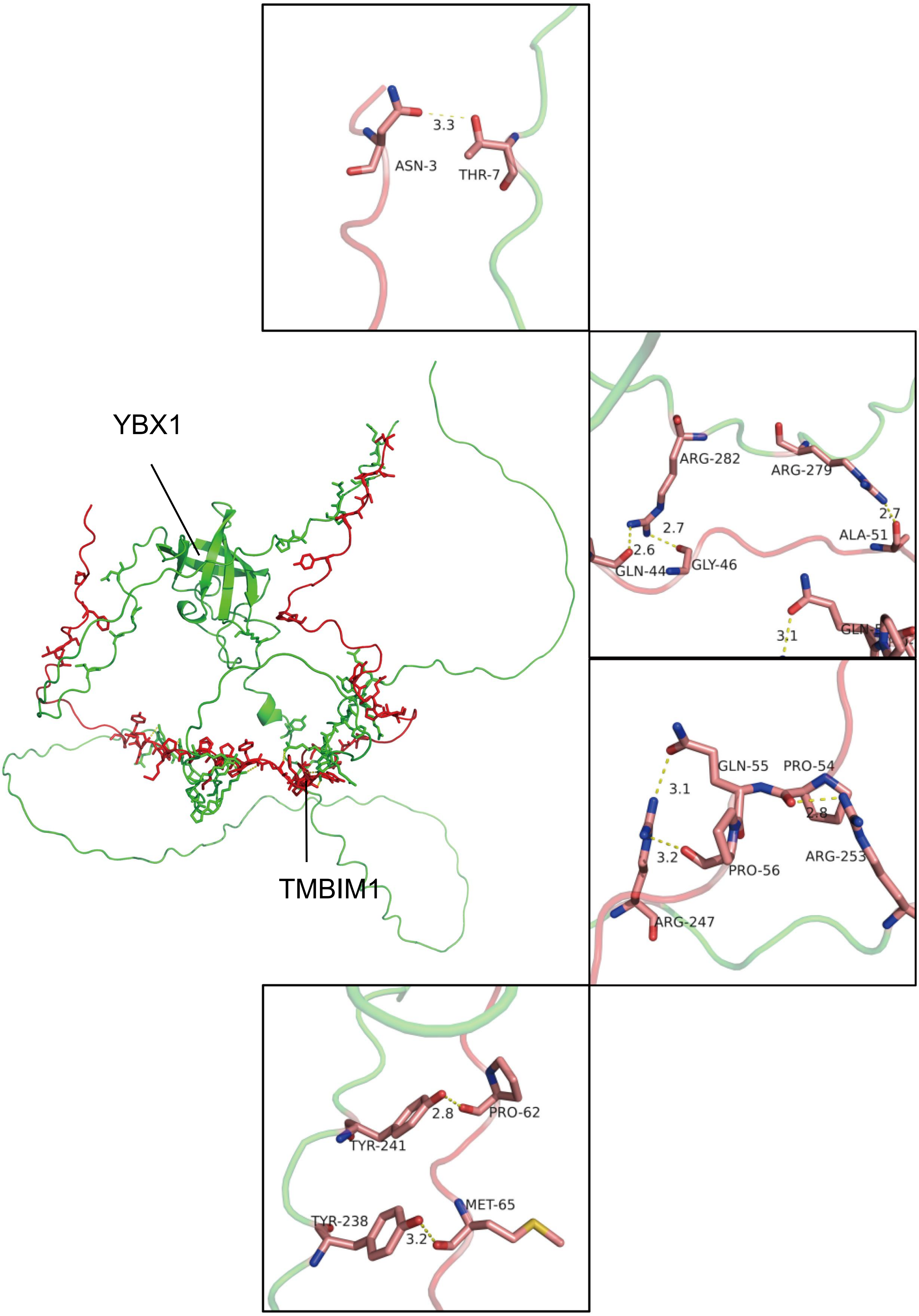

Structural interaction between YBX1 and TMBIM1.

Figure S10

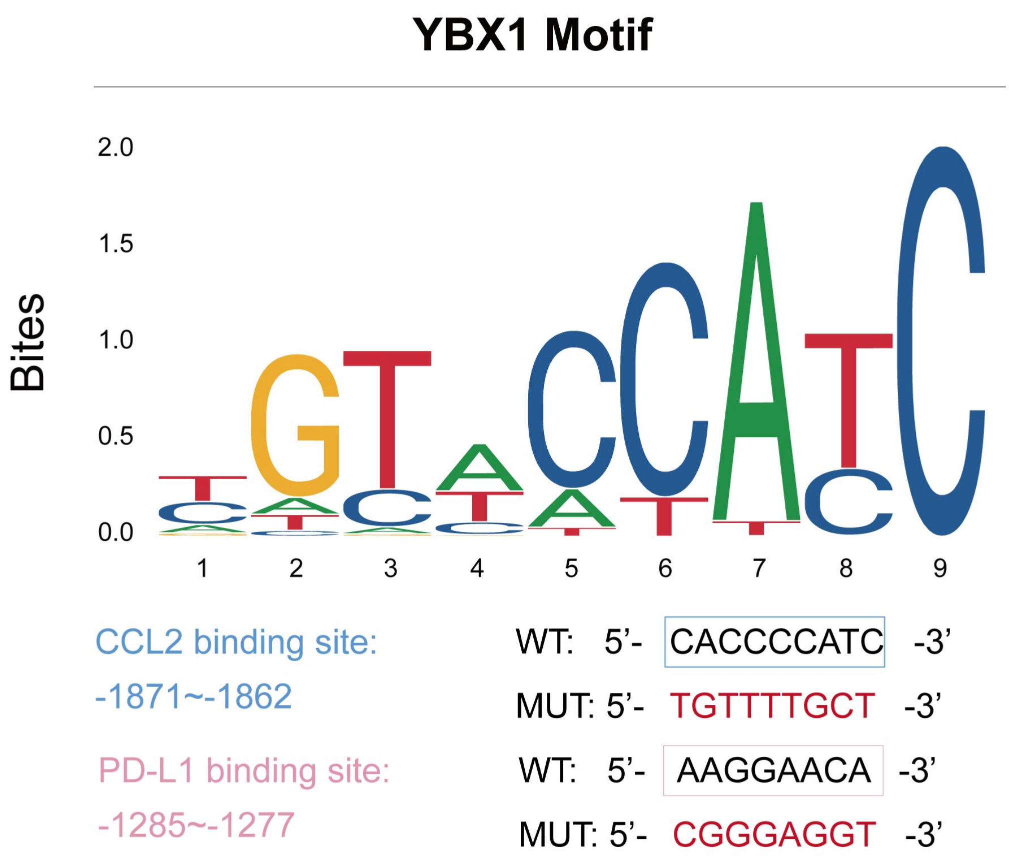

Schematic representation of the YBX1 binding motif in the CCL2 and PD-L1 promoter regions. Wild-type (WT) and mutant (MUT) sequences for the CCL2 (-1871 to -1862) and PD-L1 (-1286-1277) binding sites are shown.

Figure S11

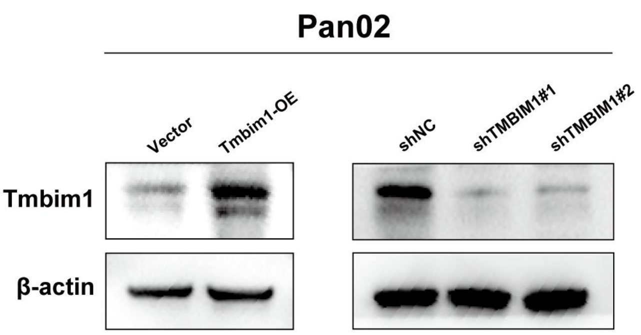

Western blot analysis of TMBIM1 expression in Pan02 cells. Left panel: Tmbim1-OE compared to vector control. Right panel: TMBIM1 knockdown using two independent shRNAs (shTMBIM1#1 and shTMBIM1#2) compared to the negative control (shNC). β-Actin serves as the loading control for all experiments.

Figure S12

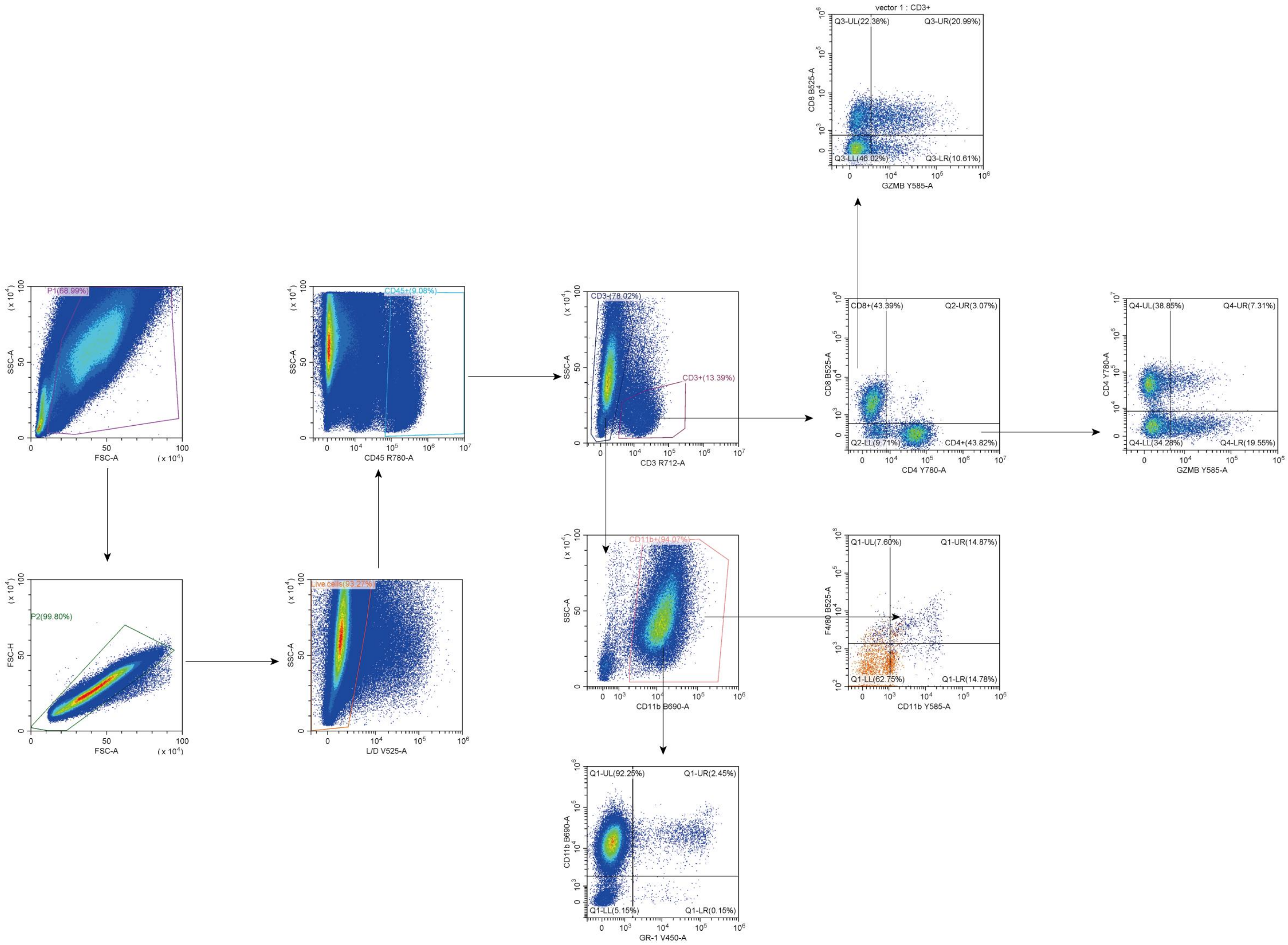

Figure S12. Flow cytometry gating strategy for immune cell analysis.

The gating strategy of multiple immune cells ($CD8^+$ T cells, $CD4^+$ T cells, MDSCs, Macrophages). The frequency of tumor-infiltrating immune cells in tumor bearing mice were used by flow cytometry.

Figure S13

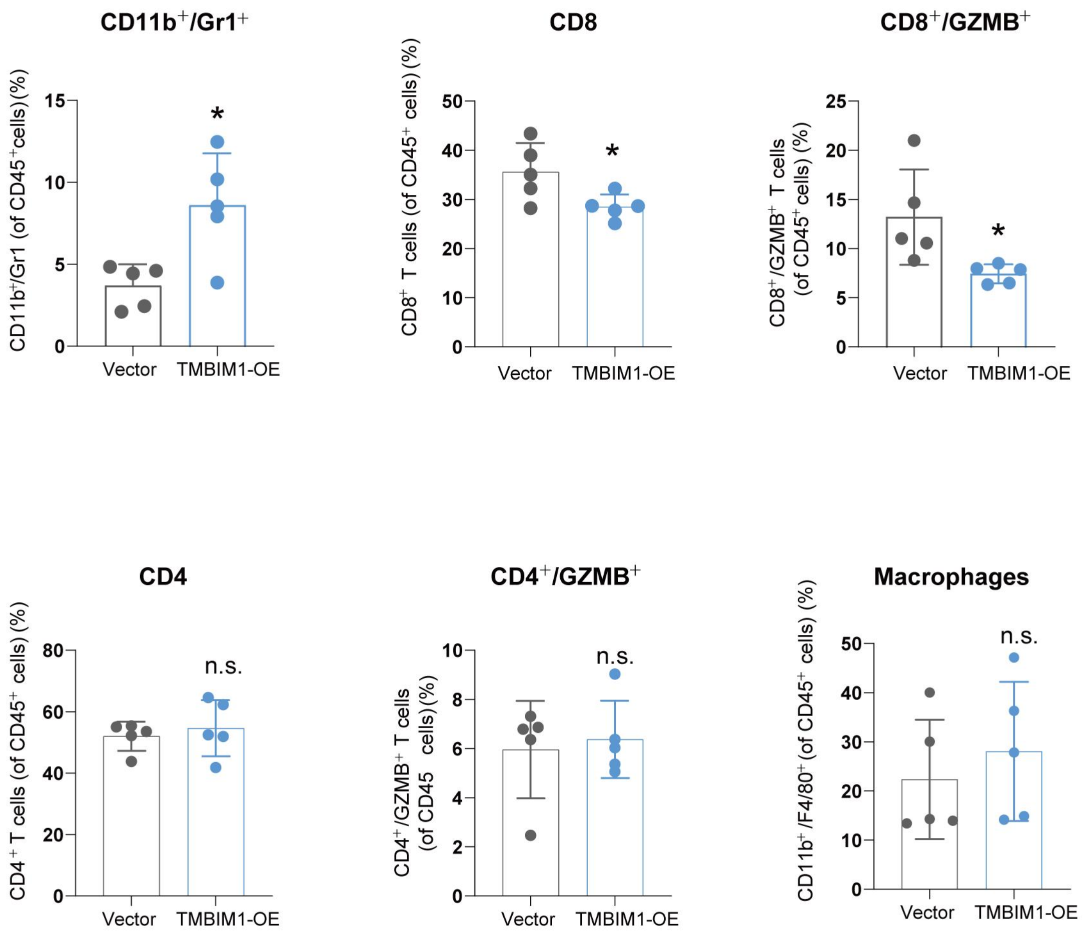

Immune cell profiling in Vector control and TMBIM1 overexpression groups.

This figure shows immune cell markers in two groups: Vector and TMBIM1 overexpression. The upper three panels represent immune cells with significant differences between the two groups: $CD11b^+GR1^+$ cells, $CD8^+$ T cells, $CD8^+/GZMB^+$ T cells; The lower three panels show immune cells with no significant differences between the two groups: $CD4^+$ T cells, $CD4^+GZMB^+$ T cells, $CD11b^+F4/80^+$ macrophages, with no statistical difference observed. Statistical analysis for all comparisons was performed using a Student's t-test.

Figure S14

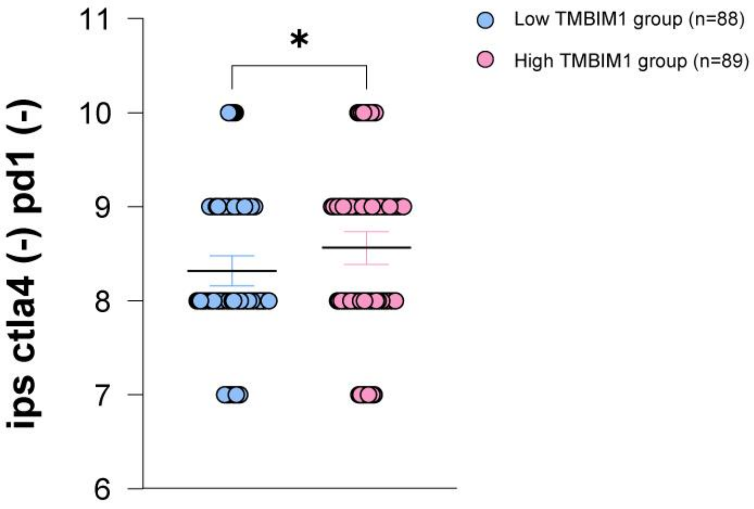

Comparison of ips CTLA-4(-) + PD-1(-) scores in low and high TMBIM1 expression groups based on TCIA databases.

This figure shows the comparison of low TMBIM1 and high TMBIM1 expression groups with respect to IPS CTLA-4 (-) and PD-1 (-) status, using data from the TCIA database. Significant differences were observed between the 2 groups. Statistical analysis was performed using a Student's t-test.

Figure S15

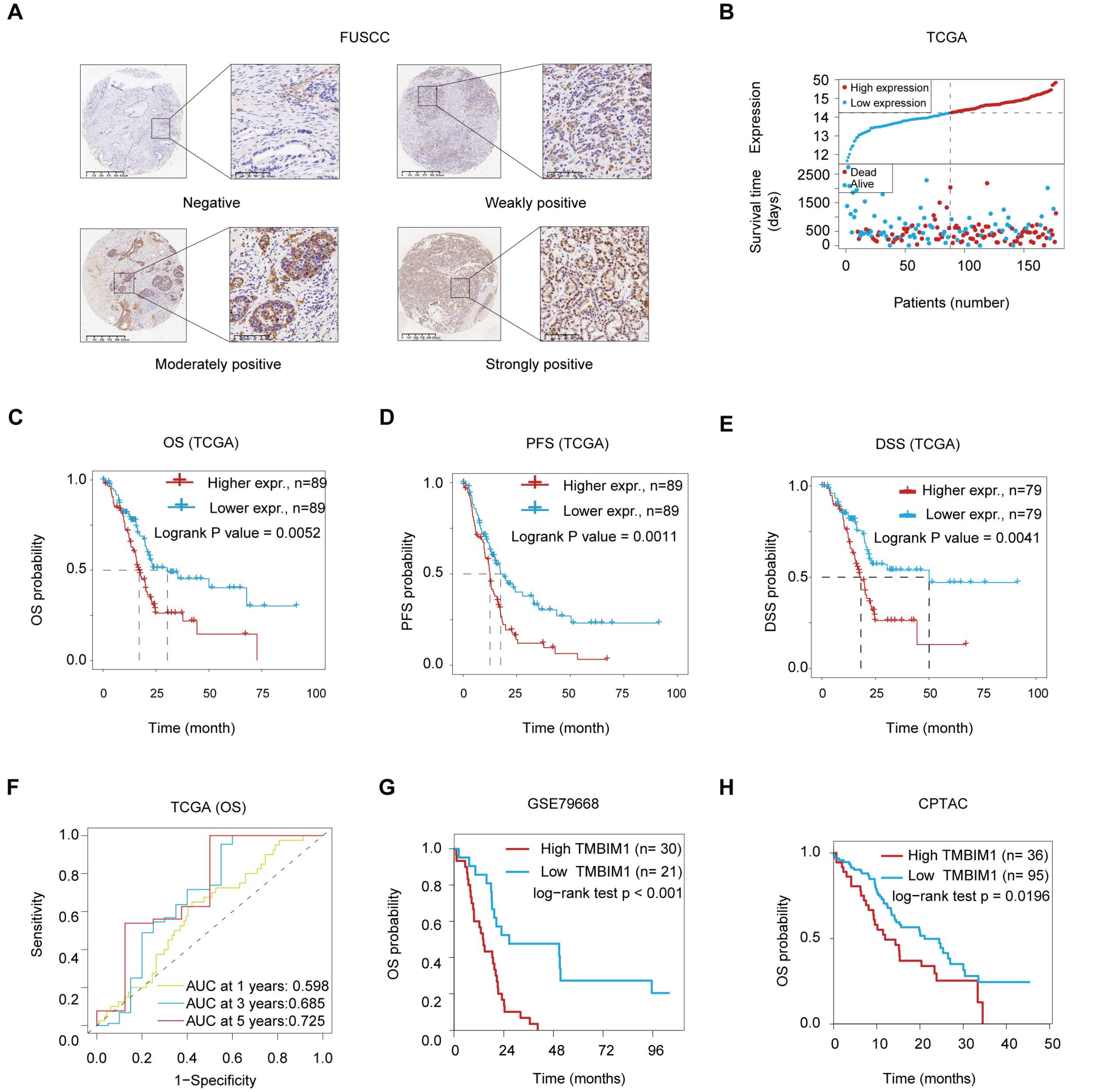

Expression and survival analysis of TMBIM1 in pancreatic cancer patients.

(A) Immunohistochemical staining of TMBIM1 in pancreatic cancer tissues from the FUSCC cohort showing various levels of expression: negative, weakly positive, moderately positive, and strongly positive. (B) Scatter plot of TMBIM1 expression levels in the TCGA-PAAD cohort, stratified by survival time, demonstrating a correlation between higher TMBIM1 expression and shorter survival. (C) Kaplan-Meier survival curve showing OS probability in the TCGA cohort, with higher TMBIM1 expression associated with significantly poorer OS (Log-rank P = 0.0052). (D) Kaplan-Meier survival curve showing PFS

probability in the TCGA cohort, indicating worse PFS in patients with higher TMBIM1 expression (Log-rank P = 0.0011). (E) Kaplan-Meier survival curve for DSS in the TCGA cohort, revealing that higher TMBIM1 expression correlates with significantly poorer DSS (Log-rank P = 0.0041). (F) ROC curves for 1-, 3-, and 5-year OS prediction based on TMBIM1 expression in the TCGA cohort, with AUC values of 0.598, 0.685, and 0.725, respectively. (G) Kaplan-Meier survival curve of OS probability in the GSE79668 dataset, demonstrating that higher TMBIM1 expression is significantly associated with reduced OS (Log-rank P < 0.001). (H) Kaplan-Meier survival curve of OS probability in the CPTAC dataset, showing significantly shorter OS in patients with higher TMBIM1 expression (Log-rank P = 0.0196).
